# Increasing JAK/STAT Signaling Function of Infant CD4^+^ T Cells during the First Year of Life

**DOI:** 10.3389/fped.2017.00015

**Published:** 2017-02-21

**Authors:** Myra Grace dela Peña-Ponce, Jennifer Rodriguez-Nieves, Janice Bernhardt, Ryan Tuck, Neelima Choudhary, Michael Mengual, Katie R. Mollan, Michael G. Hudgens, Sigal Peter-Wohl, Kristina De Paris

**Affiliations:** ^1^Department of Microbiology and Immunology, School of Medicine, University of North Carolina, Chapel Hill, NC, USA; ^2^Division of Neonatal Perinatal Medicine, Department of Pediatrics, School of Medicine, University of North Carolina, Chapel Hill, NC, USA; ^3^Lineberger Cancer Center, Center for AIDS Research, University of North Carolina, Chapel Hill, NC, USA; ^4^Gillings School of Global Public Health, Center for AIDS Research, University of North Carolina, Chapel Hill, NC, USA

**Keywords:** infant CD4^+^ T cell development, cytokines, JAK/STAT signaling, STAT activation, age-dependent changes

## Abstract

Most infant deaths occur in the first year of life. Yet, our knowledge of immune development during this period is scarce and derived from cord blood (CB) only. To more effectively combat pediatric diseases, a deeper understanding of the kinetics and the factors that regulate the maturation of immune functions in early life is needed. Increased disease susceptibility of infants is generally attributed to T helper 2-biased immune responses. The differentiation of CD4^+^ T cells along a specific T helper cell lineage is dependent on the pathogen type, and on costimulatory and cytokine signals provided by antigen-presenting cells. Cytokines also regulate many other aspects of the host immune response. Therefore, toward the goal of increasing our knowledge of early immune development, we defined the temporal development of the Janus kinase (JAK)/signal transducers and activators of transcription (STAT) signaling function of CD4^+^ T cells using cross-sectional blood samples from healthy infants ages 0 (birth) to 14 months. We specifically focused on cytokines important in T cell differentiation (IFN-γ, IL-12, and IL-4) or in T cell survival and expansion (IL-2 and IL-7) in infant CD4^+^ T cells. Independent of the cytokine tested, JAK/STAT signaling in infant compared to adult CD4^+^ T cells was impaired at birth, but increased during the first year, with the most pronounced changes occurring in the first 6 months. The relative change in JAK/STAT signaling of infant CD4^+^ T cells with age was distinct for each cytokine tested. Thus, while about 60% of CB CD4^+^ T cells could efficiently activate STAT6 in response to IL-4, less than 5% of CB CD4^+^ T cells were able to activate the JAK/STAT pathway in response to IFN-γ, IL-12 or IL-2. By 4–6 months of age, the activation of the cytokine-specific STAT molecules was comparable to adults in response to IL-4 and IFN-γ, while IL-2- and IL-12-induced STAT activation remained below adult levels even at 1 year. These results suggest that common developmental and cytokine-specific factors regulate the maturation of the JAK/STAT signaling function in CD4^+^ T cells during the first year of life.

## Introduction

Every day, 17,000 children under the age of 5 years die with close to 50% of these deaths being caused by infectious diseases (1). Most infant deaths occur in the first year of life (1), at a time when the infant has to simultaneously acquire the ability to tolerate normal commensal flora and to recognize pathogenic stimuli. To adapt and overcome this challenge, the infant immune response is qualitatively distinct from that of adults. Yet, most of our knowledge of infant immunity is derived from CB analysis only. Thus, an important gap remains in our understanding of the kinetics and factors that drive and regulate the functional maturation of infant immune cells postnatally.

The development of the infant’s immune system is shaped by many different factors that range from genetic predispositions to nutrition, pathogen exposure, environmental stimuli, and others [reviewed in Ref. (2)]. It would be an insurmountable task to address all these factors adequately in a single study. The current study focused on CD4^+^ T cells because they play a key role in innate, in cellular, and in humoral adaptive immunity by secreting cytokines that (i) activate monocytes/macrophages and dendritic cells, (ii) promote effector function of CD8^+^ T cells, and (iii) influence antibody maturation and isotype switching (3–10). Increased susceptibility and severity of infectious diseases in infants has been attributed to impaired CD4^+^ T helper (Th) 1 responses. The bias of infant CD4^+^ T cells toward T helper 2 (Th2) responses was suggested more than 25 years ago in mice (11), and human studies confirmed reduced IFN-γ production by infant CD4^+^ T cells (12–15).

The underlying immune mechanisms that contribute to the infant’s Th2 bias have only recently been discovered. Cytokines play a major role in deciding the fate of naïve CD4^+^ T cells toward the development of a specific Th cell lineage (16–21). The production of the Th1-defining cytokine IFN-γ by CD4^+^ T cells is critically dependent on IL-12 (22–24). However, not only do infant antigen-presenting cells produce less IL-12 than adults (12, 14, 25–29), naïve CD4^+^ T cells, that represent the majority of neonatal T cells, lack the expression of the IL-12Rβ2 (30–33). The preferential induction of IL-4, the prototype Th2 cytokine, versus IFN-γ in infant CD4^+^ T cells is further regulated by epigenetic modulations. Thus, the IL-12p35 gene is in a repressive chromatin state in infant dendritic cells (34, 35), and while the promoter of IFN-γ is hypermethylated in infant compared to adult CD4^+^ T cells (36, 37), gene loci associated with Th2 cytokines, such as IL-13, are hypomethylated (38).

In addition to Th cell differentiation, cytokines regulate many other immune processes (17, 39–45). Cytokines encompass a large group of diverse soluble biological mediators that can act in an autocrine, paracrine, or endocrine fashion to exert their functions. Aberrations in cytokine signaling and/or gene deficiencies have been associated with serious clinical outcomes, such as severe immunodeficiency (46, 47), inflammatory immune disorders (48), and altered lymphopoiesis (49). The importance of cytokine signaling in host immunity is further underlined by the fact that several viruses have evolved to inhibit specific components of cytokine signaling pathways to limit the induction of antiviral immunity (50, 51).

Cytokines mediate their specific function through the activation of distinct tyrosine kinases after receptor ligation. Cytokines binding to their receptors cause receptor dimerization and activation of Janus (JAK) kinases that can then recruit specific transcription factors, the signal transducers and activators of transcription (STAT) (52, 53). This signaling pathway was first described in response to interferons (53). There are four members in the JAK kinase family and seven mammalian STATs. Therefore, the specificity of cytokine function is achieved in a stepwise fashion, through activation of distinct JAK kinases, the subsequent formation of specific homo- or heterodimers of activated STATs, and finally the regulated expression of distinct genes upon translocation of the STAT molecules to the nucleus (52, 53).

Considering the key role of cytokines in many aspect of host immunity and disease, the current study defined the temporal development of JAK/STAT signaling function of CD4^+^ T cells in response to cytokines important in Th cell differentiation (IFN-γ, IL-12, and IL-4) and in T cell expansion (IL-2 and IL-7) using cross-sectional blood samples from healthy infants ages 0 (birth) to 14 months. Our analysis was focused on key components of the JAK/STAT signaling pathway: (i) cytokine receptor expression, (ii) JAK activation, and (iii) the activation of cytokine-specific STAT molecules. IFN-γ and IL-12 signal through JAK1/JAK2 and JAK2/Tyk2, respectively, while IL-4, IL-2, and IL-7, as members of the common gamma chain (γc) cytokine family, all signal through JAK1 and JAK3 [reviewed in Ref. (54)]. As the JAK1 kinase is common to all of the signaling pathways, the activation of specific STAT molecules by distinct cytokines represented a key aspect of our analysis. Thus, to assess age-dependent changes in IFN-γ, IL-12, or IL-4-specific signaling, we measured the activation of STAT1, STAT4, or STAT6, respectively. To assess IL-2- and IL-7-mediated cytokine signaling, STAT5 activation was measured (54). Furthermore, to address a potential role of JAK/STAT signaling in Th-2 biased infant immune responses, we compared the activation of JAK2 and STAT1 by IFN-γ to the activation of JAK3 and STAT6 in response to IL-4.

## Materials and Methods

### Human Subjects

Study subjects were recruited from the University of North Carolina, Chapel Hill (UNC-CH) and the NC Children’s Hospital. CB samples from full-term infants were collected immediately after birth or obtained from the Carolinas Cord Blood Bank. Direct capillary or venous blood samples (0.5–3.0 mL) from infants age 2 weeks to 14 months were collected by nursing or phlebotomy personnel during routine physician visits. The infants consisted of similar numbers of males and females. The exact number of infants per age group is listed in Table 1; we had 70 CB samples, 35 samples from infants between the ages of 1 and 9 months and 64 samples from 1 year olds (10–14 months). The large number of infants (*n* = 169) was required because limited blood volumes (0.5–2.0 mL) from infants age 2 weeks to 1 year restricted the number of assays and parameters that could be analyzed. The infant participants were recruited from the North Carolina population that consists of about 46% Caucasians, 46% African-Americans, and 8% other groups, and within the ethnic categories, 10% represent Hispanics.

**Table 1:** **Study population**.

Age	No. of subjects by sex			
	Male	Female	Unknown	Total
Birth	24	24	22^a^	70
1–3 months	3	7	0	10
4–6 months	6	11	2	19
7–9 months	2	4	0	6
10–14 months	39	24	1	64
Total	74	70	25	169

*^a^Cord blood samples obtained from the Carolinas Cord Blood Bank*.

Exclusion criteria included HIV-positive status of the mother, known *in utero* infections, treatment of the mother with immunosuppressive drugs, diagnosis of mother or child with immunosuppressive disorder, life-threatening malformations of the infant or life expectancy <6 months. Infant blood samples were also excluded if the infant had a bleeding disorder or had a chronic infection. The Virology, Immunology, and Microbiology Core of the UNC Center for AIDS Research provided blood samples from healthy adults. Age, sex, and race of the adult donors were unknown. The study was approved by the UNC-CH Institutional Review Board, and informed parental consent was obtained. Institutional guidelines strictly adhere to the World Medical Association’s Declaration of Helsinki.

### Sample Processing

Cord blood from full-term infants was collected into CB collection bags containing CPD anticoagulant, whereas all other blood samples were collected into EDTA-containing blood tubes. Blood samples were processed within 24 h. Whole blood was used for Phosflow™ analysis of JAK2 and STAT proteins; for all other assays, peripheral blood mononuclear cells (PBMCs) were isolated by gradient density separation using Lymphocyte Separation Medium (MP Biomedicals, OH) as described (55, 56).

### CD4^+^ T Cell Enrichment

CD4^+^ T cells were enriched by magnetic bead separation using the CD4^+^ T cell enrichment Kit from Stemcell Technologies (Vancouver, BC, Canada) according to the manufacturer’s instructions. The purity of CD4^+^ T cell populations was confirmed by flow cytometry and exceeded 96%.

### Cytokine Receptor Expression

Peripheral blood mononuclear cells were stained with CD3 (SP34-2)-Pacific Blue, CD4 (L200)-PerCPCy5.5, CD8 (RPA-T8)-Alexa Fluor700, CD45RA (5H9)-FITC, and CCR7 (3D12)-PeCy7 to determine naïve [T(N): CD45RA^+^CCR7^+^], central memory [T(CM): CD45RA^−^CCR7^+^], and effector/effector memory cell [T(Eff/EM), CD45RA^±^CCR7^−^] T cell populations. All antibodies, unless noted otherwise, were purchased from BD Biosciences (San Jose, CA, USA). The expression of cytokine receptors on CD4^+^T cells was quantitated using PE-labeled antibodies: IFN-γR1 (CD119-GIR-208), IFN-γR2 (2HUB-159, Biolegend, San Diego, CA, USA), IL-4Rα (hIL4R-M57), IL-2Rα (CD25-clone-4E3, Miltenyi Biotec, Auburn, CA, USA), or IL-2Rβ (CD122-clone-Mikβ). Cytokine receptor expression is reported as percentage of CD4^+^ T cell populations or quantitated on a per cell basis using a standard curve generated with Quantibrite PE beads (BD Biosciences). The staining protocol was performed at 4°C. Samples were acquired on a LSRII instrument (BD Biosciences) by collecting a minimum of 300,000 events. The flow cytometry data were analyzed using FlowJo Software (Tree Star, Ashland, OR, USA), version 9.8. Antibodies were titrated prior to use and gating was based on Fluorescence Minus One (FMO) and isotype antibody controls.

### Phosflow™ Analysis

Phosflow™ analysis was performed to measure the activation of JAK2 after IFN-γ stimulation or the activation of STAT proteins according to the manufacturer’s protocols (BD Biosciences) using Perm Buffer III (CD4^+^ T cells) or Perm Buffer IV (NK cells). Although most JAK and STAT molecules contain multiple tyrosine and/or serine phosphorylation sites, the analysis was limited to a single phosphorylation site. Briefly, 300–400 µL of whole blood was treated for 10 min with 10 µg/mL of human recombinant IFN-γ (Miltenyi Biotec) and samples were then stained with antibodies to total JAK2 or with antibodies to phosphorylated JAK2 (Tyr1008; clone D4A8, Cell Signaling Technology, Danvers, MA, USA). Phospho-JAK2 (pJAK2) was detected with anti-rabbit IgG (H + L), F(ab’)2 Fragment (Alexa Fluor 647, Cell Signaling Technology). To detect the phosphorylation of specific STAT proteins after stimulation with IFN-γ IL-4, IL-2, or IL-7, whole blood was incubated for 15 min with 10 µg/mL of recombinant IFN-γ, 0.1 µg/mL of recombinant IL-4 (R&D, Minneapolis, MN, USA) or 0.1 µg/mL human IL-2 or IL-7 (Miltenyi Biotec), respectively, and subsequently stained for phosphorylated STAT-1 (Tyr 701; clone 58D6, Cell Signaling Technology), STAT6 (Y641; clone pY641) or STAT5 (Y694; clone pY694, both BD Biosciences), respectively. Non-phosphorylated STAT1, STAT6, or STAT5 proteins were measured in parallel. Unstimulated whole blood served as negative control. STAT4 phosphorylation (pSTAT4 Y693) was measured using the same protocol, except that PBMCs were stimulated for 45 min with IL-12 (75 ng/mL) in serum-free RPMI 1640 media. To distinguish CD4^+^ T cells with different antigen experience, blood samples were costained with CD3 (SP34-2)-PeCy7, CD4 (L200)-PerCPCy5.5, and CD27 (L128)-PE, and naïve, central memory, and effector/effector memory populations were defined as being CD27^+^, CD27^low^, or CD27^−^, respectively (57). Cytokine concentrations and incubation times were determined in preliminary experiments (data not shown). Samples were acquired on a LSRII instrument analyzed with FlowJo Software. Cells positive for JAK2 or STAT phosphorylation are reported as percentage of CD4^+^ T cells after subtracting the values from the untreated samples. Antibodies were titrated prior to use and gating was based on FMO and isotype antibody controls. In addition, a subset of samples was analyzed on the Amnis^®^ ImageStreamX instrument according to the manufacturer’s protocol.

### JAK3 Activation

The phosphorylation of JAK3 in response to stimulation with IL-4 or IL-2 was measured by Western blot. CD4^+^ T cells were enriched as described above and either stimulated immediately with IL-4 or IL-2 or preactivated for 2 days prior to cytokine stimulation. For the latter, CD4^+^ T cells were stimulated at a concentration of 2–4 × 10^6^ cells/mL with plate-bound anti-CD3 (10 µg/mL; clone SP34-2), precoated overnight in a 24-well plate, and cultured at 37°C and 5% CO_2_. Controls were set up in parallel with media only. After 48 h, to activate JAK3, CD4^+^ T cells were stimulated for 5 min with 0.1 µg/mL recombinant human IL-4 or IL-2 at 37°C in a water-bath followed by a 5-min centrifugation at 300 × *g* (total stimulation time: 10 min). Cells were lysed with 100 µL of Blue Loading Pack (Cell Signaling Technology) per 4 × 10^6^ cells. Protein concentrations were determined using the Pierce 660 nm Assay (Thermo Scientific, Rockford, IL, USA). Samples were loaded onto 8% Novex^®^ Tris-Glycine gels (Novex^®^, Life Technologies, NY, USA). The XCell SureLock™ Mini Cell was used for electrophoresis in Novex^®^ Tris-Glycine SDS running buffer and for subsequent transfer using Novex^®^ Tris-Glycine transfer buffer. Transfer PVDF membranes were obtained from Bio-Rad Laboratories (Hercules, CA, USA). All detection antibodies and reagents were obtained from Cell Signaling Technology unless noted otherwise. To determine JAK3 phosphorylation, the membranes were incubated with phospho-JAK3 antibody (Tyr980/981; clone D44E3; 1:1,000 dilution) in 1× TBST overnight, stripped using Restore Plus Western blot (Pierce/Thermo Scientific, Rockville, IL, USA) and re-probed for non-phosphorylated JAK3 (clone D1H3; 1:1,000 dilution:) and beta (β)-Actin (clone 13E5; 1:50,000 dilution) as loading control. The membranes were then incubated with secondary anti-Rabbit IgG HRP-linked antibody at a dilution of 1:2,000 and developed using the Amersham™ ECL Prime Western Blotting Detection Reagent (GE Healthcare Life Sciences, Pittsburgh, PA, USA). Images were captured using the Bio-Rad ChemiDoc MP System and densitometric measurements were obtained using the Image Lab Software Version 5.2.1. Protein sizes were verified based on the NuPage HiMark Prestained Protein Standard (Life Technologies, Grand Island, NY, USA).

### Statistical Analysis

Data were analyzed using GraphPad Prism Software (Version 6, 2014; La Jolla, CA, USA). Continuous measurements were compared between independent age groups using non-parametric Mann–Whitney tests. Statistical comparisons were only performed if the sample size was *n* ≥ 4 in both age groups. Associations between continuous variables was estimated using non-parametric Spearman rank correlation. To visualize the age-dependent changes in STAT activation in response to IL-4, IFN-γ, and IL-2, a LOESS trend curve was generated with a smooth parameter of 0.5. Only statistically significant *p*-values are shown in the figures. A 0.05 significance level was used with no adjustment for multiple hypothesis testing.

## Results

### Cytokine Receptor Expression on Infant CD4^+^ T Cells

Cytokine receptor expression was evaluated both by quantifying the number of receptors per CD4^+^ T cell and by determining the percentage of CD4^+^ T cells expressing each cytokine receptor. Two main comparisons were performed. First, we determined whether there was a significant change in cytokine receptor expression from birth to 1 year of age. Second, we tested whether the expression of cytokine receptors differed between 1-year-old infants and adults. These comparisons were conducted both for total CD4^+^ T cells and for CD4^+^ T cell subsets. The analysis of CD4^+^ T cells according to their phenotypic characterization as naïve [T(N); CD45RA^+^CCR7^+^], central memory [T(CM); CD45RA^−^CCR7^+^], or effector memory/effector [T(EM/Eff); CD45RA^±^CCR7^−^] CD4^+^ T cells was important because the majority of infant peripheral blood CD4^+^ T cells represent naïve CD4^+^ T cells and the relative frequencies of these CD4^+^ T cell population change with age and pathogen exposure. Thus, this analysis was necessary to accurately evaluate age-related differences in infant CD4^+^ T cells from birth to 1 year, and equally critical for the comparison of infant and adult CD4^+^ T cell parameters.

Cord blood CD4^+^ T cells expressed significantly more IL-4Rα molecules per CD4^+^ T cell than adults (Table 2). Although there was an age-dependent decline in median IL-4Rα numbers/CD4^+^ T cell comparing birth to 1 year, these differences were not statistically significant. The percentage of IL-4Rα^+^CD4^+^ T cells also did not vary substantially with age (Table 3). To initiate signaling upon IL-4 binding, the IL-4Rα chain dimerizes with the common γ-chain (γc); we did, however, not measure the common γ-chain, because previous studies have already shown that it is expressed at lower levels in infants (58).

**Table 2:** **Cytokine receptor expression**.

Cytokine receptor	Cell type	Median number of cytokine receptors/CD4^+^ T cell (range)						Birth versus 10–14 months	10–14 months versus adult
		Birth	1–3 months	4–6 months	7–9 months	10–14 months	Adult		
IL-4Rα	CD4^+^ T	409 (61–1,026)	407 (300–412)	311 (271–429)	303 (271–451)	286 (201–662)	278 (179–368)	NS*	NS
	CD4^+^ T (N)	440 (59–1,106)	433 (350–434)	381 (368–505)	314 (273–380)	283 (196–996)	281 (210–423)	NS	NS
	CD4^+^ T (CM)	324 (57–910)	360 (252–362)	247 (230–350)	275 (250–459)	287 (196–331)	265 (172–391)	NS	NS
	CD4^+^ T (EM/Eff)	449 (83–1,040)	391 (315–568)	450 (265–567)	516 (397–725)	333 (175–395)	304 (200–371)	NS	NS

IFN-γR1	CD4^+^ T	1,118 (138–4,738)	2,019 (1,018–5,247)	972 (785–1,119)	n.d.**	834 (627–1,388)	653 (390–1,824)	NS	*p* = 0.0057
	CD4^+^ T (N)	1,202 (137–3,804)	2,350 (1,172–13,874)	1,387 (885–1,638)	n.d.	944 (668–2,756)	872 (430–3,293)	NS	NS
	CD4^+^ T (CM)	1,276 (140–5,039)	2,477 (1,015–4,238)	883 (783–946)	n.d.	776 (599–1,159)	718 (364–2,063)	NS	NS
	CD4^+^ T (EM/Eff)	783 (133–12,727)	926 (777–1,687)	708 (608–817)	n.d.	640 (594–909)	568 (392–1,792)	NS	NS

IFN-γR2	CD4^+^ T	686 (105–1,641)	974 (894–1,072)	1,527 (813–1,940)	n.d.	630 (299–1,062)	635 (271–2,018)	NS	NS
	CD4^+^ T (N)	750 (101–2,102)	1,581 (1,155–1,826)	2,525 (1,210–3,842)	n.d.	860 (413–1,367)	890 (332–2,213)	NS	NS
	CD4^+^ T (CM)	564 (71–1,696)	688 (627–906)	853 (551–965)	n.d.	451 (253–856)	507 (223–2,573)	NS	NS
	CD4^+^ T (EM/Eff)	624 (151–7,201)	995 (473–1,379)	806 (548–1,547)	n.d.	684 (355–3,409)	584 (289–1,873)	NS	NS

IL-2Rα	CD4^+^ T	696 (214–2,420)	1,548 (1,369–1,606)	1,052 (621–1,780)	1,446 (1,032–1,633)	1,121 (807–1,815)	501 (200–1,439)	*p* = 0.0074	*p* = 0.0015
	CD4^+^ T (N)	610 (158–3,166)	1,836 (1,407–1,912)	1,382 (678–2,821)	2,005 (1,782–2,155)	1,575 (1,019–2,497)	420 (238–1,170)	*p* = 0.0006	*p* < 0.0001
	CD4^+^ T (CM)	667 (232–2,330)	1,345 (1,293–1,349)	975 (696–1,335)	1,266 (869–1,356)	981 (607–1,467)	452 (183–1,176)	*p* = 0.0378	*p* = 0.0010
	CD4^+^ T (EM/Eff)	853 (452–2,702)	1,730 (1,365–1,815)	1,122 (613–1,661)	1,469 (947–1,699)	1,176 (911–2,119)	659 (220–1,827)	NS	*p* = 0.0022

IL-2Rβ	CD4^+^ T	338 (45–1,776)	n.d.	410 (294–704)	n.d.	289 (207–427)	266 (145–461)	NS	NS
	CD4^+^ T (N)	444 (46–1,491)	n.d.	436 (377–3,302)	n.d.	300 (201–540)	340 (171–671)	NS	NS
	CD4^+^ T (CM)	261 (52–3,329)	n.d.	362 (270–393)	n.d.	272 (203–383)	225 (134–457)	NS	NS
	CD4^+^ T (EM/Eff)	344 (43–3,726)	n.d.	338 (294–519)	n.d.	280 (212–347)	265 (130–457)	NS	NS

*NS, not significant; p > 0.05; **n.d., not done.

**Table 3:** **Cytokine receptor expression**.

Cytokine receptor	Cell type	Percentage of CD4^+^ T cells expressing specific cytokine receptors (range)						Birth versus 10–14 months	10–14 months versus adult
		Birth	1–3 months	4–6 months	7–9 months	10–14 months	Adult		
IL-4Rα	CD4^+^ T	0.4 (0.1–3.8)	0.3 (0.1–0.3)	0.4 (0.2–0.4)	0.4 (0.3–0.6)	0.2 (0.1–4.0)	0.5 (0.1–1.2)	NS*	NS
	CD4^+^ T (N)	2.5 (0.2–61.3)	1.4 (0.2–4.8)	0.6 (0.6–0.7)	0.7 (0.6–0.8)	0.4 (0.1–23.8)	0.9 (0.2–4.2)	NS	NS
	CD4^+^ T (CM)	0.2 (0.0–0.7)	0.1 (0.1–0.2)	0.3 (0.1–0.3)	0.3 (0.2–0.5)	0.2 (0.1–4.8)	0.4 (0.1–1.7)	NS	NS
	CD4^+^ T (EM/Eff)	0.6 (0.1–27.0)	0.1 (0.1–0.2)	0.1 (0.1–0.3)	0.6 (0.1–0.7)	0.2 (0.0–0.6)	0.2 (0.0–0.6)	*p* = 0.0114	NS

IFN-γR1	CD4^+^ T	1.1 (0.2–3.9)	1.4 (0.8–2.5)	1.9 (1.3–3.0)	n.d.**	1.4 (0.5–3.3)	4.3 (1.3–10.0)	NS	*p* < 0.0001
	CD4^+^ T (N)	2.1 (0.3–19.1)	4.4 (0.8–5.6)	2.1 (1.7–2.8)	n.d.	1.5 (0.4–2.5)	0.8 (0.3–2.7)	NS	NS
	CD4^+^ T (CM)	0.8 (0.1–3.4)	1.0 (0.5–1.8)	1.6 (1.1–2.9)	n.d.	1.2 (0.4–2.3)	1.1 (0.3–5.6)	NS	NS
	CD4^+^ T (EM/Eff)	3.4 (0.6–18.7)	3.8 (2.9–3.8)	5.9 (1.8–6.8)	n.d.	6.3 (1.7–9.3)	12.6 (3.9–31.7)	NS	*p* < 0.0001

IFNγR2	CD4^+^ T	0.5 (0.1–7.0)	0.6 (0.4–0.7)	0.5 (0.3–0.7)	n.d.	0.5 (0.2–0.8)	0.2 (0.1–1.9)	NS	NS
	CD4^+^ T (N)	3.1 (0.2–53.6)	n.d.	2.5 (0.6–5.0)	n.d.	0.4 (0.1–1.0)	0.3 (0.1–2.3)	*p* = 0.0292	NS
	CD4^+^ T (CM)	0.2 (0.0–3.6)	n.d.	0.4 (0.1–0.5)	n.d.	0.3 (0.1–0.8)	0.1 (0.0–1.0)	NS	NS
	CD4^+^ T (EM/Eff)	1.2 (0.0–14.7)	n.d.	0.4 (0.1–0.5)	n.d.	0.2 (0.0–0.7)	0.2 (0.0–4.2)	*p* = 0.0053	NS

IL-2Rα	CD4^+^ T	4.1 (1.2–16.2)	11.3 (6.9–14.7)	9.8 (7.1–14.0)	9.6 (8.6–11.7)	9.6 (7.9–13.8)	30.6 (16.2–41.4)	*p* = 0.0006	*p* < 0.0001
	CD4^+^ T (N)	3.7 (0.5–65.2)	12.6 (5.4–15.6)	8.3 (4.4–16.4)	9.1 (9.0–10.6)	7.4 (2.9–14.7)	9.7 (4.6–23.1)	NS	NS
	CD4^+^ T (CM)	1.8 (0.3–14.9)	6.4 (5.2–11.6)	6.2 (4.7–10.6)	6.4 (4.0–7.1)	8.1 (5.3–10.3)	36.8 (21.2–53.8)	*p* = 0.0016	*p* < 0.0001
	CD4^+^ T (EM/Eff)	38.3 (0.2–87.5)	77.0 (44.4–82.5)	49.8 (29.5–66.5)	42.2 (34.0–52.0)	44.2 (36.8–58.3)	47.4 (27.3–76.4)	NS	NS

IL-2Rβ	CD4^+^ T	0.5 (0.1–7.5)	n.d.	0.2 (0.0–0.3)	n.d.	0.4 (0.1–2.7)	0.9 (0.1–4.3)	NS	NS
	CD4^+^ T (N)	0.6 (0.2–11.1)	n.d.	0.2 (0.0–0.3)	n.d.	0.2 (0.0–1.8)	0.2 (0.1–1.7)	NS	NS
	CD4^+^ T (CM)	0.2 (0.0–1.4)	n.d.	0.1 (0.0–0.2)	n.d.	0.3 (0.1–2.2)	0.7 (0.1–4.4)	NS	NS
	CD4^+^ T (EM/Eff)	3.0 (0.1–16.1)	n.d.	0.7 (0.1–1.5)	n.d.	1.9 (0.2–9.6)	1.8 (0.1–8.9)	NS	NS

*NS, not significant; p > 0.05; **n.d., not done.

**Figure 1:**
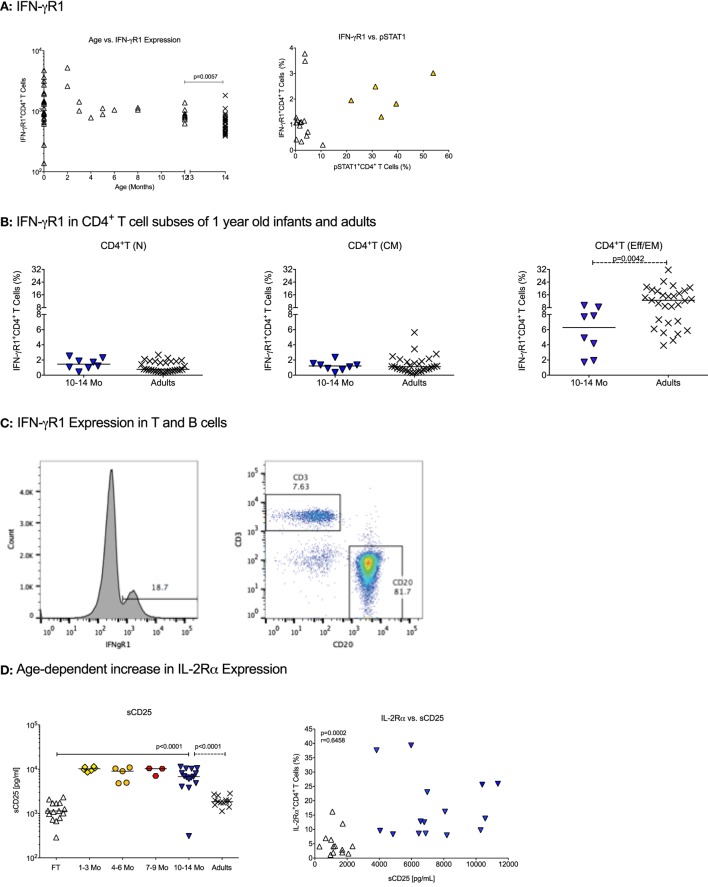
**Cytokine receptor expression in infants**. **(A)** The changes in absolute numbers of IFN-γR1 on CD4^+^ T cells and the percentage of IFN-γ^+^ CD4^+^ T cells with age. Each symbol represents an individual infant (open triangle); adults are shown by black cross symbols. The *p* values indicate statistically significant differences between 10–14 months old infants and adults. **(B)** The percentage of IFN-γ^+^ CD4^+^ T cells in naïve [T(N)], central memory [T(CM)], and effector memory/effector [T(EM/Eff)] CD4^+^ T cells of 10- to 14-month-old infants and adults. **(C)** A representative histogram of IFN-γR1-positive cells in an infant blood sample. Note that the majority of IFN-γR1-positive cells represent B cells. **(D)** The left graph shows the age-dependent increase in soluble CD25 (sCD25) plasma levels. The right graph illustrates the Spearman rank correlation between sCD25 plasma levels and IL-2Rα^+^CD4^+^ T cells using infants at birth and 1 year. Each symbol represents an individual infant, with infants at different ages being characterized by specific symbols: cord blood (CB): open triangle, 1–3 months: yellow diamonds, 4–6 months: orange circles, 7–9 months: red hexagons, 10–14 months (1 year): blue triangles. Horizontal lines represent median values. The differences in plasma sCD25 levels between CB and 1-year olds were determined by Mann–Whitney test.

The IFN-γ receptor consists of the IFN-γR1, promoting IFN-γ binding, and the IFN-γR2 that mediates signaling to the JAK kinases. The number of IFN-γR1 molecules per CD4^+^ T cell did not significantly differ between birth and 1 year (Table 2). CD4^+^ T cells in CB and in blood from 1-year-old infants expressed significantly higher numbers of IFN-γR1/CD4^+^ T cell than adults (Table 2; Figure 1A). In contrast, the percentage of CD4^+^ T cells expressing IFN-γR1 increased from birth to 1 year, and even at 1 year, the percentage of IFN-γR1^+^CD4^+^ T cells was significantly lower compared to adults (Table 3, Figure 1A). This difference was especially pronounced in the subset of effector memory/effector CD4^+^ T cells (Table 3; Figure 1B). We did not observe significant changes in total expression levels of IFN-γR2 (Table 2), and although the frequencies of naïve and effector memory/effector IFN-γR2 CD4^+^ T cells seemed to decrease in the first year, the biological significance of the changes is questionable given their very low frequencies (Table 3). Note that the relatively low frequencies of IFN-γR1 or IFN-γR2 CD4^+^ T cells in infants were not the result of poor staining, as IFN-γR-positive B cells could be easily enumerated in the same samples (Figure 1C). The analysis of the ratio of relative expression levels of IL-4Rα to IFN-γR1 or IL-4Rα to IFN-γR2 on infant CD4^+^ T cells did not reveal changes that would support a switch from a more Th2-biased response at birth versus a more balanced Th2/Th1 response at 1 year.

An analysis of the IL-2R was included because T cell expansion is a critical step in T cell differentiation. The IL-2R consists of three distinct chains, the α, β, and γ-chain. The IL-2Rα chain (or CD25) is of special importance as it is a marker of regulatory T cells (Tregs), which occur in higher frequencies in infant compared to adult blood, but CD25 is also expressed on activated T cells that are more frequent in adult peripheral blood. Both, absolute numbers of IL-2Rα per CD4^+^ T cell and the percentage of IL-2Rα^+^CD4^+^ T cells were higher at 1 year of age than at birth (Tables 2 and 3; Figure 1D). At 1 year, the number of IL-2Rα molecules per CD4^+^ T cells exceeded that of adult CD4^+^ T cells (Table 2), whereas the percentage of IL-2Rα^+^CD4^+^ T cells was significantly lower compared to adults (Table 3). Whether or not these results reflected the dichotomy of the CD25 molecule as a marker of Treg or activated CD4^+^ T cells was not a focus of the study. However, soluble CD25 (sCD25) plasma levels were higher at 1 year compared to CB (Figure 1D), and there was a significant positive correlation between plasma sCD25 levels and the percentage of IL-2Rα^+^CD4^+^ T cells (Figure 1D). Although these results were indicative of increasing activated CD4^+^ T cells with age, higher sCD25 plasma levels (Figure 1D) and IL-2Rα^+^CD4^+^ T cells (Table 3) were already increased by 1–3 months and did not further increase from 3 to 14 months.

The expression levels of IL-2Rβ did not differ between infant CD4^+^ T cells at birth and or at 1 year, or between CD4^+^ T cells of 1-year-old infants and adults. We did not measure the expression of the common γ-chain (see above).

The results highlight that naïve, central memory, and effector memory CD4^+^ T cells show age-dependent differences in cytokine receptor expression that are likely to influence the functional capacity of these CD4^+^ T cell subsets.

### IL-4 Signaling Ability of Infant CD4^+^ T Cells

As the Th2-bias of infant CD4^+^ T cells is well documented, we hypothesized that CB CD4^+^ T cells would activate the JAK/STAT signaling pathway in response to IL-4 as effectively as adult CD4^+^ T cells. Upon binding of IL-4 to its receptor, JAK1 and JAK3 will be activated by engaging the IL-4Rα or the common γ chain, respectively. As the common γ chain is also part of the IL-2 receptor, we chose to measure JAK3 activation to maximize the number of parameters that could be analyzed considering the small sample volumes available. The limited blood volumes for 10- to 14-month-old infants also required that we pooled PBMCs from multiple infants (pool 1: *n* = 9; pool 2: *n* = 5) to enrich CD4^+^ T cells for Western blot analysis.

**Figure 2:**
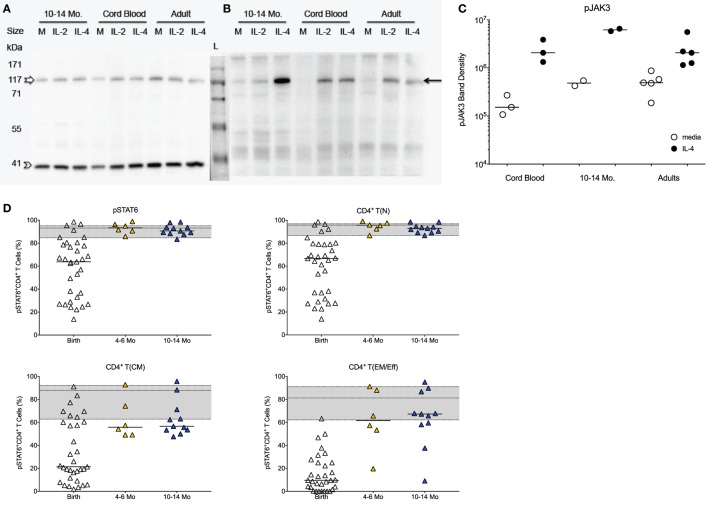
**JAK/STAT activation in infant CD4^+^ T cells in response to IL-4**. **(A)** Representative Western blot gel for total JAK3 (upper band; empty white arrow) and the β-actin control (lower band, empty arrow head) using CD4^+^ T cell protein lysates from cord blood samples, 10- to 14-month-old infants and adults stimulated with IL-4 or IL-2 in comparison to media (M) only. **(B)** Representative Western blot showing phosphorylated JAK3 (pJAK; black arrow). **(C)** Western blot gels were analyzed by densitometry to determine relative changes in JAK3 activation in media or IL-4 stimulated CD4^+^ T cells. **(D)** The graphs show the frequencies of pSTAT6^+^ cells in total CD4^+^ T cells and in naïve [T(N)], central memory [T(CM)], and effector memory/effector [T(EM/Eff)] CD4^+^ T cell populations of at birth (*n* = 33), infants aged 4–6 (*n* = 6) and 10–14 (*n* = 11) months. The gray area in each graph represents the median frequencies (middle line) of pSTAT6^+^ CD4^+^ T cells in adults (*n* = 25), with the lower and upper line corresponding to the 25th and 75th percentile. Each symbol represents an individual subject, with the exception of **(B)**: the samples for 10- to 14-month-old infants represent pools of nine or five subjects each. Statistical significant differences between two age groups were determined by non-parametric Mann–Whitney test.

The protein levels of total JAK3 were comparable between CD4^+^ T cells isolated from CB, 1-year olds or adults (Figure 2A). With the caveat that exact frequencies of pJAK3^+^CD4^+^ T cells could not be quantified by Western blot, CD4^+^ T cells isolated from CB or 1-year-old infants were equally able to activate pJAK3 in response to IL-4 (Figures 2B,C). Thus, densitometry analysis showed no statistically significant differences in the increase of median pJAK3 densities in IL-4 stimulated compared to unstimulated CD4^+^ T cells in CB (19-fold; range: 4.9- to 25.4-fold), samples of 1 year olds (13-fold; range: 10.7- to 15.4-fold), and adults (2.5-fold; range: 2.2- to 11.6-fold).

The effective activation of pJAK3 by IL-4 in CB was confirmed by the fact that the downstream IL-4-specifc transcription factor STAT6 was activated in 64% (median value) of CB CD4^+^ T cells, although subject-to-subject variation at birth was high (range: 14–99%; Figure 2D). Despite the seemingly potent induction of the JAK/STAT signaling pathway by IL-4 in CB, median frequencies of pSTAT6^+^CD4^+^ T cells were lower compared to adults (93%; range: 60–98%). The activation of pSTAT6 significantly increased with age (Figure 2D), and by 4–6 months of age, pSTAT6 activation was comparable to 1-year-old infants and to adults (Figure 2D). The age-dependent increase in IL-4-induced pSTAT6 activation observed in the total CD4^+^ T cell populations appeared to be mainly reflective of changes in naive CD4^+^ T cells (Figure 2D). Although memory CD4^+^ T cells also responded with higher pSTAT6 activation by 4–6 months and at 1 year, pSTAT6 activation remained below [T(CM)] or within the lower range [T(EM/Eff)] of adult memory CD4^+^ T cells (Figure 2D). However, as naïve cells represented the majority of infant CD4^+^ T cells, the reduced pSTAT6 activation by infant memory cells was masked in the total CD4^+^ T cell population.

**Figure 3:**
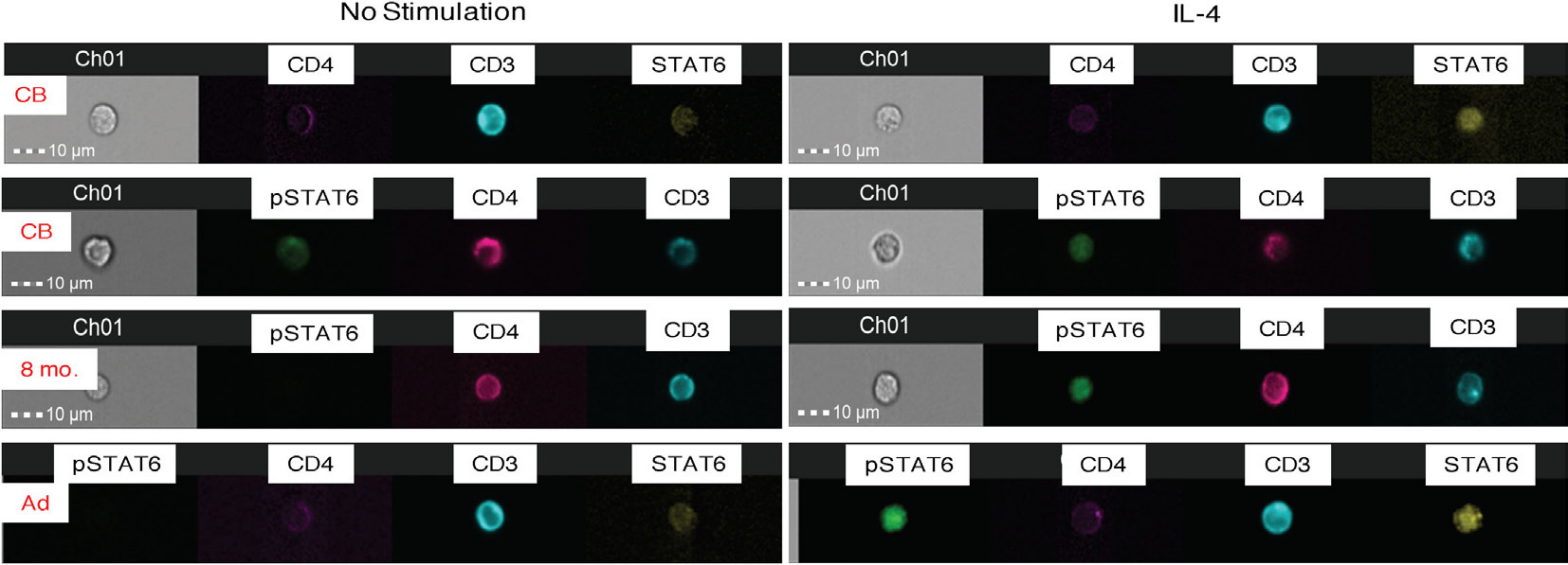
**Amnis^®^-ImageStreamX analysis of STAT6 activation**. The images are representative examples of whole blood samples that were untreated (left panels) or stimulated with IL-4 for 15 min (right panels) and then stained and analyzed for total (unphosphorylated) STAT6 and activated pSTAT6. Each image row shows a single cell labeled with CD3 (blue), CD4 (pink), unphosphorylated STAT 6 (yellow), or phosphorylated STAT6 (pSTAT6; green). The first two rows represent examples of cord blood (CB) stained with STAT 6 or pSTAT6; followed by an 8-month old infant blood sample, and one adult donor blood. Channel 1 (“Ch1”) shows the brightfield image of the cell in relation to the 10-μm size marker.

To test for the translocation of activated pSTAT6 to the nucleus, a critical event for downstream gene induction, we utilized Amnis^®^ ImageStreamX analysis. CB and adult CD4^+^ T cells showed similar expression of unphosphorylated STAT6 (Figure 3). Upon IL-4-stimulation, activated pSTAT6 was clearly detectable in the nucleus of CB CD4^+^ T cells, but the fluorescence intensity was slightly stronger in CD4^+^ T cells of an 8-month-old infant and in adult CD4^+^ T cells (Figure 3).

### Age-Dependent Increase in IFN-γ-Induced JAK/STAT Signaling

**Figure 4:**
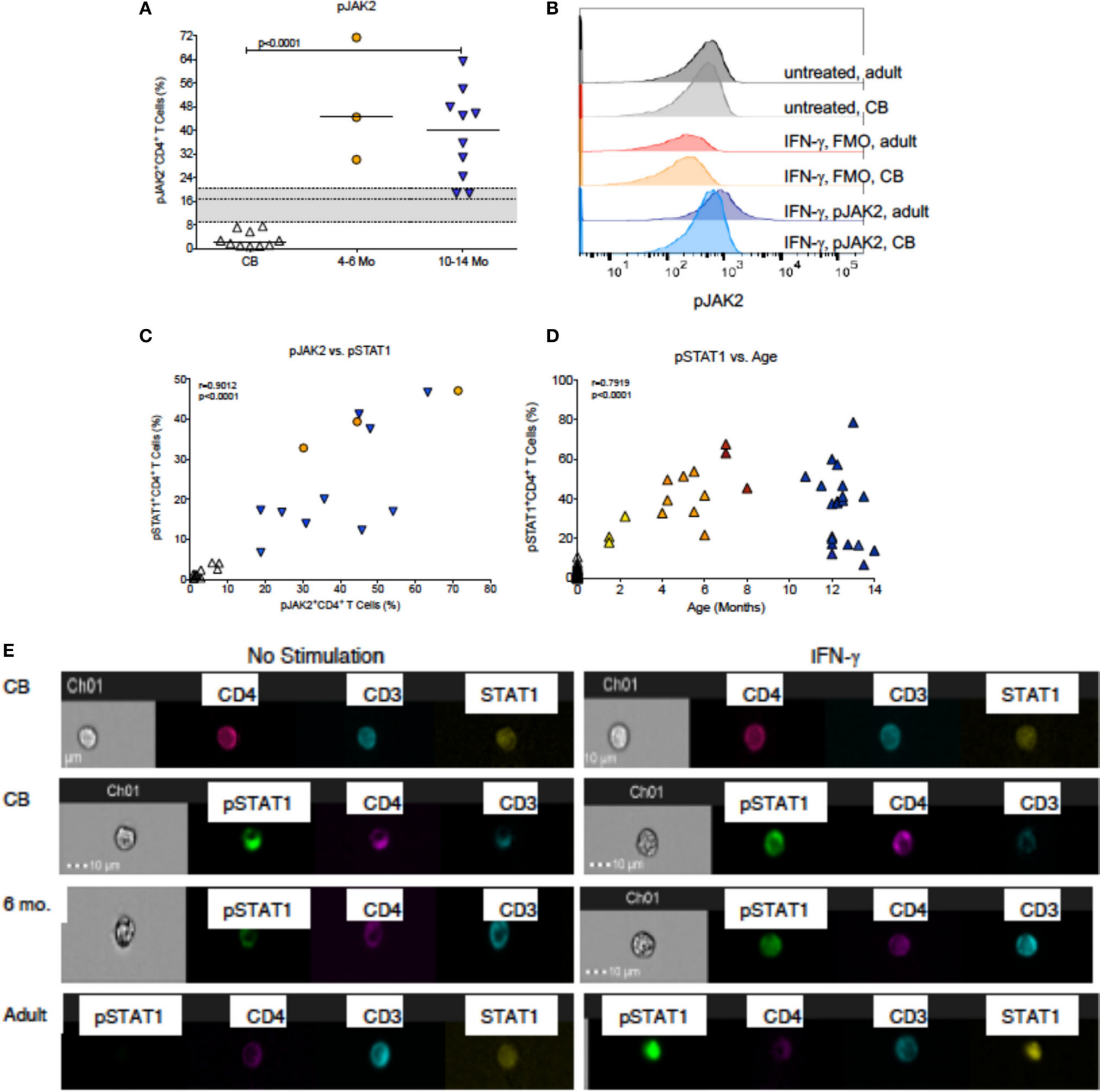
**IFN-γ-induced JAK/STAT signaling**. **(A)** The percentages of pJAK2^+^CD4^+^ T cells in cord blood (CB, *n* = 10), 4- to 6-month-old (*n* = 3), and 10- to 14-month-old (*n* = 10) infants. Median frequencies of adult (*n* = 12) pJAK2^+^CD4^+^ T cells with the 25th and 75th percentile are indicated by the gray shaded area. **(B)** Representative pJAK2 histograms from a CB sample and an adult blood donor. Note that baseline pJAK2 levels are similar in CB and adult blood, and in IFN-γ stimulated samples, the FMO controls also do not differ between CB and adult blood. In contrast, there is an induction of pJAK2 in adult, but notCB, CD4^+^ T cells after IFN-γ stimulation. **(C)** The association between pJAK2^+^ and STAT1^+^ CD4^+^ T cells in relation to age through 14 months. Note that the analysis only included infant samples that had sufficient volume to measure both parameters. **(D)** The associations between pSTAT1^+^CD4^+^ T cells and age through 14 months. Correlations were determined by Spearman rank test. **(E)** Representative examples of CB, 6-month-old infant and adult blood samples analyzed by Amnis^®^-ImageStreamX for the nuclear localization of STAT1 and pSTAT1 in unstimulated samples (left panels) or after IFN-γ stimulation (right panels). The legend is as described in Figure 3.

In contrast to JAK3 activation by IL-4, only a small fraction of CB CD4^+^ T cells induced JAK2 phosphorylation in response to IFN-γ stimulation (Figure 4A). The histograms in Figure 4B are representative for CB and adult CD4^+^ T cells and demonstrate that unstimulated CD4^+^ T cells in both age groups have similar low levels of pJAK2 and similar background level staining in FMO controls, but clearly differ in the levels of pJAK2 after IFN-γ stimulation. However, by 1 year of age, pJAK2 activation was even higher than in adult CD4^+^ T cells (Figure 4A). To more specifically determine when infant CD4^+^ T cell acquired improved IFN-γ signaling capacity, we measured STAT1 phosphorylation in CD4^+^ T cells from birth to 1 year. As expected, the increase in pSTAT1 activation paralleled the observed increase in pJAK2^+^ CD4^+^ T cells (Figure 4C). In CB, only 1.3% (median; range: 0.0–10.7%) CD4^+^ T cells activated pSTAT1 upon IFN-γ stimulation (Figure 4D). Activation of pSTAT1 was already increased by 1–3 months of age (median: 26.1%) and by 4–6 months pSTAT1^+^CD4^+^ T cell frequencies were detected in about 40% CD4^+^ T cells (Figure 4D); representative histograms are shown in Figure S1 in Supplementary Material. Although there was a significant positive correlation between increasing infant age and higher frequencies of pSTAT1^+^CD4^+^ T cells, this relationship did not appear to be linear (Figure 4D). In fact, IFN-γ-induced pSTAT1 activation seemed to plateau at about 6 months of age (Figure 4D; Table S1 and Figure S2 in Supplementary Material). As was shown for total STAT6 expression, unphosphorylated STAT1 could be detected in both CB and adult CD4^+^ T cells (Figure 4E). However, consistent with the age-dependent increase in pSTAT1 activation, activated pSTAT1 was localized mainly in the cytoplasm in CB CD4^+^ T cells, whereas in samples from 6-month-old infants pSTAT1 was clearly detectable in the nucleus (Figure 4E).

**Figure 5:**
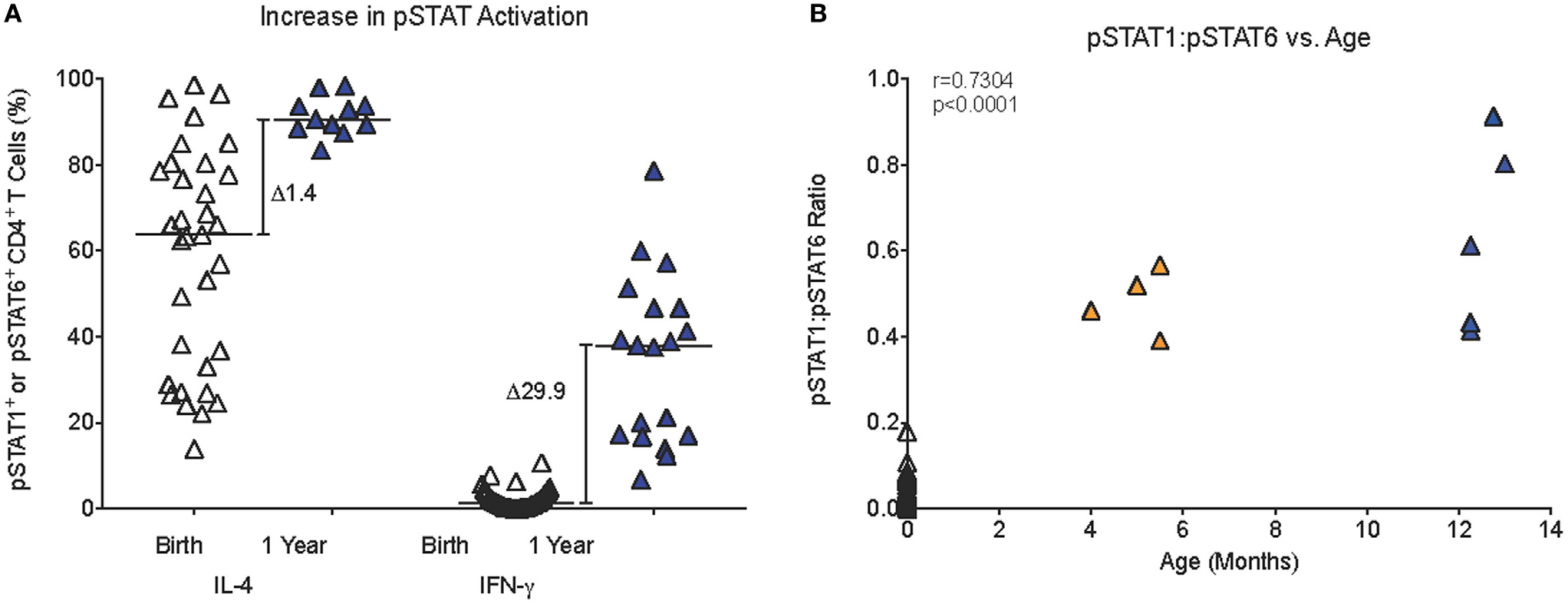
**Age-dependent changes in JAK/STAT signaling**. **(A)** The relative increase (Δ) in median pSTAT6 or pSTAT1 activation in infant CD4^+^ T cells from birth to 1 year. **(B)** A positive correlation (Spearman rank test) between infant age and the pSTAT1:pSTAT6 ratio in infant blood samples that had sufficient blood volume to analyze both pSTAT1 and pSTAT6 activation in response to IFN-γ or IL-4 stimulation, respectively. Each symbol represents an individual infant; infants of different ages are represented by distinct color symbols as described in Figure 1.

**Figure 6:**
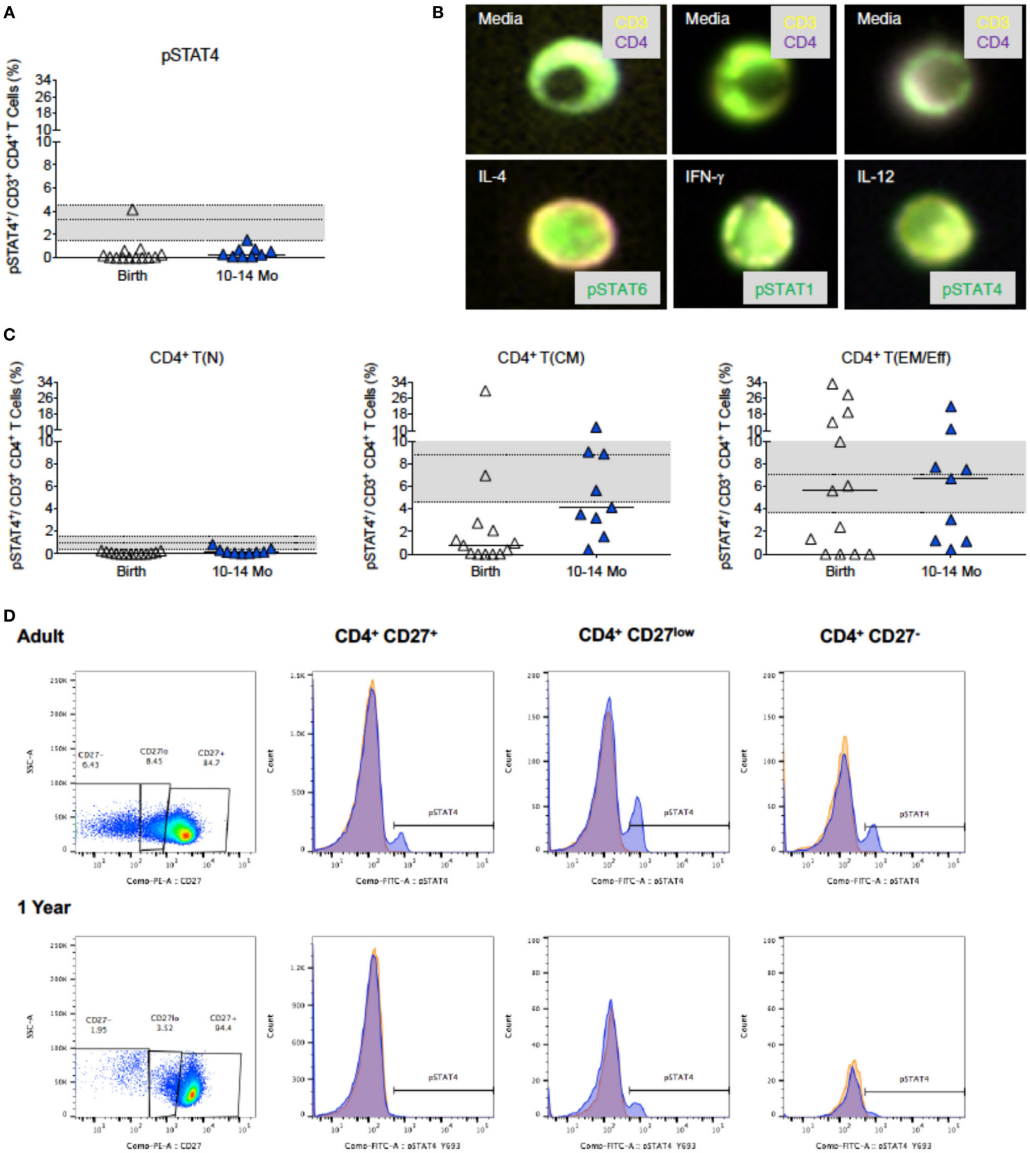
**IL-12-induced pSTAT4 activation**. **(A)** The increase in pSTAT4 activation after IL-12 stimulation of CD4^+^ T cells at birth (*n* = 18) and 1 year (*n* = 17); adult values (*n* = 27) are indicated by the gray shaded area with median, 25th, and 75th percentile. **(B)** Representative composite images of CD4^+^ T cells from 6-month-old infants stimulated with media only (top row), or with (bottom row) IL-4, IFN-γ, or IL-12 and analyzed by Amnis^®^-ImageStreamX for the nuclear localization of their relevant transcription factor. Note the distinct nuclear localization of pSTAT6 and pSTAT1 that fills almost the whole nucleus in comparison to only partial pSTAT4 nuclear localization. **(C)** is analogous to **(A)**, but shows the induction of pSTAT4 in T(N), T(CM), and T(EM/Eff) CD4^+^ T cells. The legend and the statistical comparisons are as described in Figures 1 and 2. **(D)** The relative frequencies of naïve [CD27^+^, T(N)], central memory [CD27^low^, T(CM)], and effector memory/effector [CD27^−^, T(EM/Eff)] CD4^+^ T cell populations of a representative adult and 1-year-old infant and the corresponding histograms of pSTAT4 activation after IL-12 stimulation (blue). pSTAT4 frequencies in unstimulated controls are shown by orange histograms.

Compared to only a 1.4-fold increase in median frequencies of pSTAT6^+^CD4^+^ T cells from birth (63.8%) to 1 year (90.6%), median frequencies of pSTAT1^+^CD4^+^ T cells increased 30-fold in the first year (birth: 1.3%; 1 year: 38.0%) (Figure 5A). Furthermore, in samples for which sufficient blood volume was available to test both IL-4 and IFN-γ-induced JAK/STAT signaling, we observed a more than 10-fold increase in the ratio of pSTAT1:pSTAT6 from 0.04 (median; range: 0.0–0.2) at birth to 0.5 (range: 0.4–0.9) at 1 year (Figure 5B). Whether or not these findings have direct implications for CD4^+^ Th cell differentiation, was outside the scope of the current study. To further probe CB CD4^+^ T cells for reduced signaling function in response to Th1-promoting cytokines, we tested whether STAT4 phosphorylation after *in vitro* IL-12 stimulation was also reduced. Indeed, only 0.42% (median value; range: 0.0–4.2%) CB CD4^+^ T cells responded with STAT4 phosphorylation (Figure 6A). Despite higher IL-12 signaling ability of CD4^+^ T cells in 1-year-old infants, median frequencies of pSTAT4^+^CD4^+^ T cells still accounted for <1% in 10- to 14-month-old infants (range: 0.1–1.7%) and remained significantly lower compared to pSTAT4^+^ frequencies in adult CD4^+^ T cells (median: 3.3%; range: 0.2–18.2%) (Figure 6A). Consistent with this result, few CD4^+^ T cells with nuclear pSTAT4 localization could be detected in 1-year-old infants (Figure 6B). In addition, it appeared that the nuclear translocation might have been less efficient compared to nuclear localization of pSTAT6 or pSTAT1 in CD4^+^ T cells of 1-year-old infants stimulated with IL-4 or IFN-γ, respectively (Figure 6B). However, the tools for a more quantitative protein analysis of the Amnis^®^ImageStreamX data were not available.

Considering that the IL-12Rβ2 chain is not expressed on naive CD4^+^ T cells (59, 60), we compared in pSTAT4^+^CD4^+^ T cell frequencies in memory CD4^+^ T cells of different age groups. Despite the low frequencies of CD4^+^ T cells that can be phenotypically characterized as T(CM) or T(EM/Eff) CD4^+^ T cells in CB, about 1 and 4%, respectively, could activate pSTAT4. By 1 year, the functional capacity of T(EM/Eff) CD4^+^ T cells to signal in response to IL12 was equivalent to adult T(EM/Eff) CD4^+^ T cells (Figures 6C,D), but the low frequencies of differentiated infant CD4^+^ T cells likely prevented the detection of this response within the total CD4^+^ T cell population (Figure 6A). Despite higher frequencies of memory CD4^+^ T cells in adults, IL-12Rβ2 expression is relatively low within the total CD4^+^ T cell population (Figure S3 in Supplementary Material). In contrast, NK cells (CD3^−^CD14^−^CD19^−^CD56^+^), even in infants, show more pronounced expression of IL-12Rβ2 (Figure S3 in Supplementary Material), with IL-12Rβ2 expression being highest on CD16^+^ NK cell subsets (Figure S3 in Supplementary Material).

### JAK/STAT Signaling by Infant CD4^+^ T Cells in Response to the γ Chain Cytokines IL-2 and IL-7

**Figure 7:**
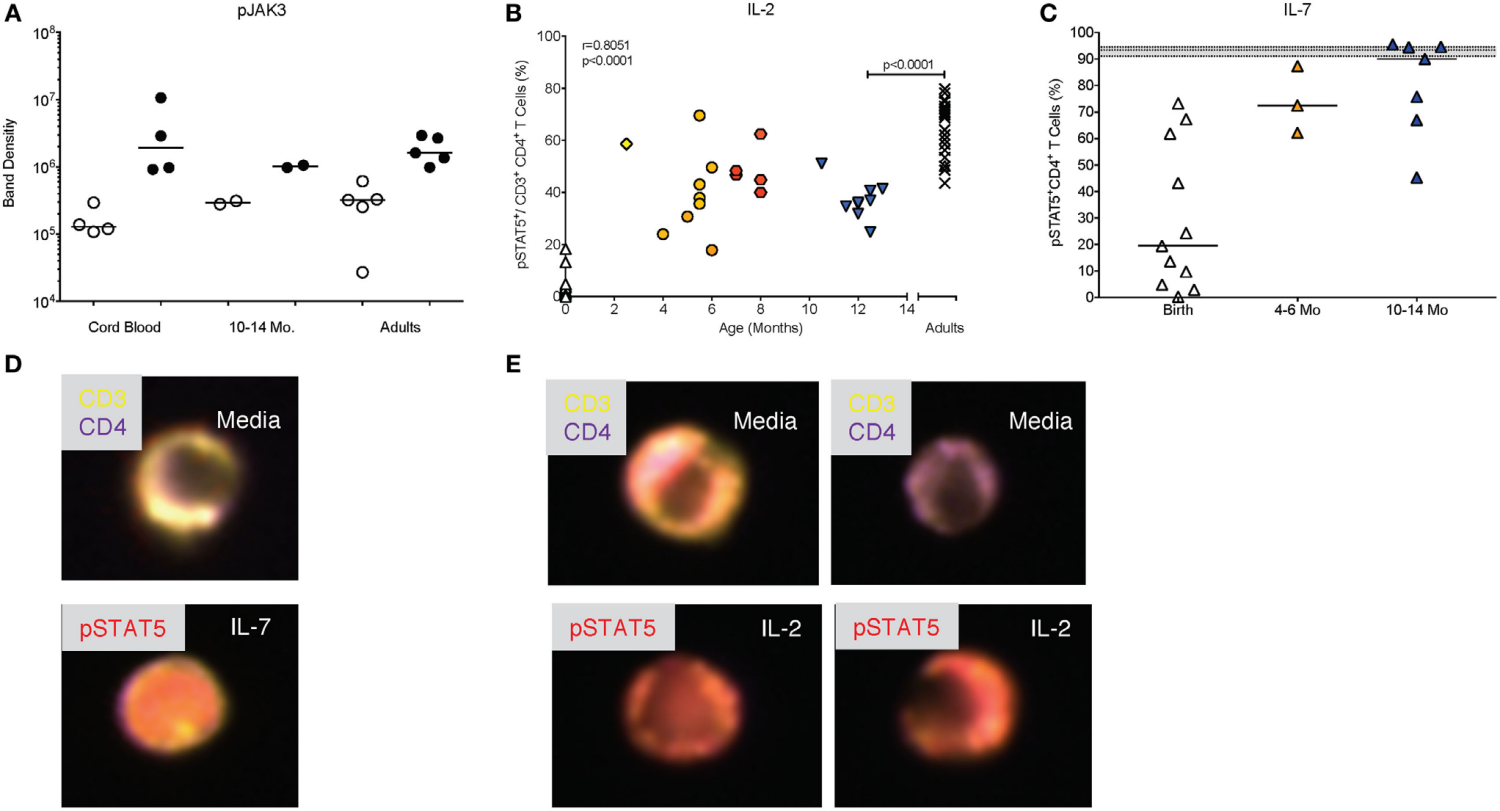
**Differential activation of pSTAT5 by IL-2 and IL-7**. **(A)** Western blot analysis of pJAK3 activation as described in Figure 2C. **(B)** The age-dependent change in pSTAT5^+^ CD4^+^T cells from birth to 14 months of age with Spearman rank correlation values. Adult values are shown in comparison and were compared to 10- to 14-month-old infants by Mann–Whitney test. **(C)** Frequencies of pSTAT5^+^ CD4^+^T cells in response to *in vitro* IL-7 stimulation are shown for cord blood (*n* = 11), 4- to 6-month-old (*n* = 3), and 10- to 14-month-old (*n* = 7) infants. The gray area indicates median frequencies of adult pSTAT5^+^ CD4^+^T (*n* = 17) with borders representing the 25th and 75th percentiles. Each symbol represents an individual subject. Horizontal lines represent median values. Each age group is assigned a different symbol as described in Figure 1; Mann–Whitney test was used for between group comparisons. **(D,E)** Representative composite images of CD4^+^ T cells from 6-month-old infants stimulated with IL-7 **(D)** or IL-2 **(E)** and analyzed by Amnis^®^-ImageStreamX. The response of CD4^+^ T cells to IL-2 was more variable with some cells showing pSTAT5 activation and nuclear localization (left images), whereas other cells remained unresponsive, indicated by lack of nuclear staining with pSTAT5 (right images). In contrast, the majority of CD4^+^ T cells showed pSTAT5 activation and nuclear translocation after IL-7 stimulation.

IL-2 is a key cytokine promoting the proliferation and expansion of CD4^+^ T cells. As pointed out above, IL-2 signals through the common γ chain of the IL-2R and activates the JAK3 kinase. The activation of pJAK3 by IL-2 appeared to be comparable in CD4^+^ T cells from CB, blood of 1-year-old infants and adults (Figure 7A; see Figure 2A for controls). However, because we could not quantitate the exact number of pJAK3^+^CD4^+^ T cells by Western blot (Figure 7A), we measured the activation of the IL-2 specific transcription factor STAT5, downstream of JAK3. This analysis showed that frequencies of pSTAT5^+^CD4^+^ T cells were positively correlated (*p* < 0.0001) with increasing age between birth and 1 year (Figure 7B). However, even at 1 year, median pSTAT5^+^CD4^+^ T cell frequencies (36%; range: 25–51%) remained significantly lower compared adults (median: 69%; range: 44–80%) (Figure 6B).

To determine whether this effect was specific for IL-2, we tested whether JAK/STAT signaling to the related common γ-chain cytokine IL-7, that also promotes proliferation, was impaired in infants as well. Compared to <1% median frequencies of pSTAT5^+^ CD4^+^ T cells activated by IL-2 in CB, IL-7 induced about 19% (range: 0.3–73.2%) pSTAT5^+^CD4^+^ T cells (Figure 7C). IL-7-induced frequencies of pSTAT5^+^CD4^+^ T cells increased to 72% (range: 62.1–87.3%) by 4–6 months and reached 90% (range: 45.2–95.5%) by 1 year, levels comparable to those achieved in adults (median: 93.3%; range: 83.0–97.6%) (Figure 7C). The differential signaling capacity of infant CD4^+^ T cells to IL-2 or IL-7 was further supported by image analysis of blood samples from 6-month-old infants (Figure 7D). Although we could consistently detect pSTAT5 in the nucleus of samples stimulated with IL-7, activation and nuclear translocation of pSTAT5 in response to IL-2 was variable (Figure 7E).

**Figure 8:**
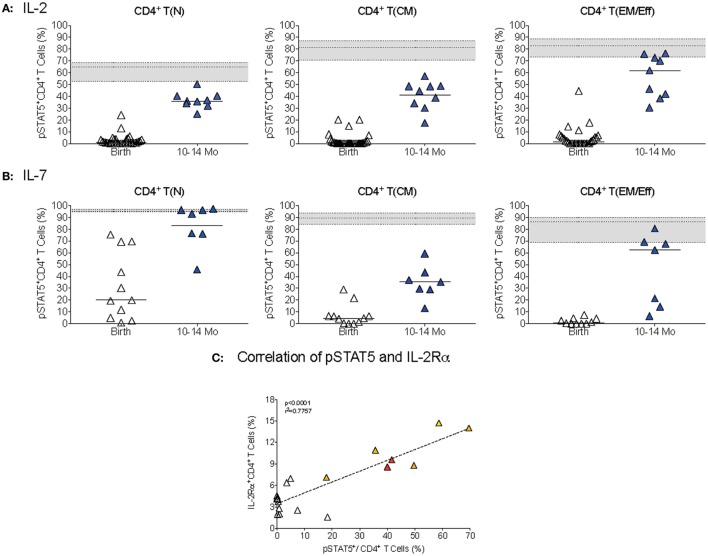
**CD4^+^ T cell differentiation in STAT5 activation**. **(A,B)** The percentages of T(N), T(CM), and T(EM/Eff) CD4^+^ T cells that were activated pSTAT5 in response to IL-2 **(A)** or IL-7**(B)**, respectively, in infant blood samples collected at birth and 1 year. Each symbol represents an individual infant; horizontal lines represent median values. The gray area indicates median frequencies of adult pSTAT5^+^ CD4^+^T cells (*n* = 17) with borders representing the 25th and 75th percentiles. **(C)** The correlation by Spearman rank test between pSTAT5^+^CD4^+^T cells and IL-2Rα^+^ CD4^+^ T cells in infant samples of different ages (colored symbols as explained in Figure 1). Note that we only included samples that were analyzed for both pSTAT5^+^ activation and IL-2Rα^+^ expression.

The difference in IL-2 versus IL-7 signaling was mainly due to differential activation of naïve CD4^+^ T cells (Figures 8A,B). At 1 year, pSTAT5^+^ frequencies within the naïve CD4^+^ T cell population were still significantly lower (*p* < 0.0001) compared to adults after IL-2 stimulation, whereas IL-7-induced pSTAT5^+^ CD4^+^ T(N) cell frequencies were comparable between 1-year olds and adults (Figure 8). In T(CM) and T(EM/Eff) CD4^+^ T cells of 1-year-old infants, the activation of pSTAT5 remained below adult levels after both IL-2 and IL-7 stimulation (Figures 8A,B). Consistent with the fact that CD4^+^ T cell differentiation into memory T cells is associated with increased expression of CD25, the IL-2Rα, we noted an age-dependent positive correlation between IL-2-induced pSTAT5 activation and frequencies IL-2Rα^+^CD4^+^ T cells (Figure 8C).

## Discussion

To develop novel prevention and intervention strategies aimed at reducing neonatal and infant morbidity and mortality, we first need to understand how the immune system develops in the healthy infant. Toward this goal, the current study defined the temporal development of the JAK/STAT signaling function in human infant CD4^+^ T cells from birth to 1 year of age in response to specific cytokines. We focused on cytokines important in Th cell differentiation, because increased susceptibility to infectious diseases in infants has been associated with Th2-biased responses.

Our results suggest that the maturation of the JAK/STAT signaling function in infant CD4^+^ T cells might be regulated by common and cytokine-specific factors. Regardless of the cytokine tested, the activation of cytokine-specific STAT molecules was significantly lower in CB CD4^+^ T cells compared to 1-year-old infants and to adults. During the first year of life, there was a statistically significant age-dependent increase in STAT activation, with the most pronounced changes occurring within the first 6 months (Table S1 and Figure S2 in Supplementary Material). The overall magnitude and the kinetics of changes in JAK/STAT signaling function, however, were distinct for each cytokine. In addition, the ability to activate the JAK/STAT pathway varied dependent on the differentiation status of CD4^+^ T cells.

As different cytokines exert distinct functions, the activation of the JAK/STAT signaling pathway will likely be regulated in a cytokine-dependent manner, too. Therefore, comparisons in the relative activation of the JAK/STAT signaling pathway by different cytokines have to consider these factors. Assuming that adult CD4^+^ T cells are fully functional, the activation of specific STAT molecules in response to distinct cytokines could be considered as 100% response rate. Applying this assumption and by expressing the activation of STAT molecules in infant CD4^+^ T cells as a function of STAT activation by the same cytokines in adult CD4^+^ T cells, we can demonstrate cytokine-dependent differences in JAK/STAT signaling function in infant CD4^+^ T cells (see Figure S4 in Supplementary Material). Among the cytokines evaluated, CD4^+^ T cells at birth showed relatively strong activation of the JAK/STAT signaling in response to IL-4 and also to IL-7, whereas the induction of the JAK/STAT pathway in response to IL-2, IFN-γ and IL-12 was less developed. The obvious question arising is which factors govern these signaling differences. We plan to define such factors in future studies; their evaluation was outside the scope of the current study as the first step was to determine how CD4^+^ T cell JAK/STAT signaling function changes from birth to 1 year in response to distinct cytokines.

The differences in the age-dependent increase in STAT activation by distinct cytokines can be partially explained by the preponderance of naïve versus more differentiated CD4^+^ T cells in infants compared to adults. This was most evident in IL-12-mediated JAK/STAT signaling. Thus, although median frequencies of IL-12-induced pSTAT4^+^ CD4^+^ T cells within the naïve CD4^+^ T cell population of CB or blood from 1-year-old infants were significantly lower than those in adult CD4^+^ T cells, median frequencies of pSTAT4^+^CD4^+^ T(EM/Eff) cells were within the range of those induced in adult CD4^+^ T cells by IL-12 (see Figure 6). However, as the majority of CD4^+^ T cells at 1 year still represent naïve CD4^+^ T cells, this response by memory CD4^+^ T cells was masked when only the total CD4^+^ T cell population was analyzed. These results emphasize the importance of comparing functional responses in relevant T cell subpopulations.

Naïve and memory CD4^+^ T cells differ in the expression of specific cytokine receptor chains. Yet, the formation of the fully assembled cytokine receptor, which consists of multiple distinct chains, is required to induce the JAK/STAT signaling pathway. Thus, the differences in pSTAT4 activation between naïve and memory CD4^+^ T cells can be explained, at least partially, by the lack of the IL-12Rβ2 chain on naive CD4^+^ T cells (59, 60). Similarly, the differential activation of pSTAT5 by IL-2 and IL-7 was likely the result of distinct expression levels of the IL-2Rα and the IL-7Rα on naïve and memory CD4^+^ T cells. The IL-7Rα is highly expressed on recent thymic emigrants that are abundant in the naïve CD4^+^ T cell population in CB (61). In contrast, naïve CD4^+^ T cells lack the expression of the IL-2Rα chain and can therefore only form the intermediate affinity IL-2R consisting of the IL-2Rβ and the common γ-chain. As the number of activated and differentiated CD4^+^ T cells increases, the high affinity IL-2R complex (IL-2Rαβγ) can be formed and pSTAT5 activation will increase concurrently. Indeed, we found a significant positive correlation between increasing frequencies of IL-2Rα^+^ and pSTAT5^+^ CD4^+^ T cell frequencies from birth to 1 year of age.

It should be emphasized though that the phenotypic characterization of CD4^+^ T cells as naïve or memory T cell was not sufficient to explain differences between CD4^+^ T cells in CB or blood samples collected from 1-year-old infants or adults. Again, this can be best illustrated when we express the activation of STAT molecules in naïve or memory infant CD4^+^ T cells as percent of adult CD4^+^ T cell responses in the same populations (Figure S5 in Supplementary Material). Thus, additional factors likely contribute to age-dependent changes in JAK/STAT signaling function between infant and adult CD4^+^ T cells.

While the current study examined JAK/STAT signaling in response to single cytokines only, in response to an *in vivo* stimulus, the cytokine milieu will be shaped by the interplay of multiple cytokines secreted by diverse cell populations. For example, IFN-γ, can promote the induction of the IL-12Rβ2 chain on naïve CD4^+^ T cells (59, 60). IL-2-induced pSTAT5 can transcriptionally regulate the expression of the IL-12Rβ and IL-4Rα (62, 63), and thereby could influence JAK/STAT signaling in response to IL-12 and IL-4. As our results show that IL-2-induced pSTAT5 activation, even at 1 year of age, is significantly lower in infant compared to adult CD4^+^ T cells, it is possible that reduced IL-2 signaling impedes the development of optimal Th1 responses in infants (Figure S4 in Supplementary Material). One could hypothesize that at birth IL-2-induced pSTAT5 would preferentially bind to the *il4ra* promoter because the *ifn*γ promoter of infant CD4^+^ T cells is hypermethylated (36, 37), but once the methylation status changes with age, pSTAT5 could also bind to the *ifn*γ and/or to the *il12r*β*2* promoters (Figure S4 in Supplementary Material). In fact, recent studies have provided evidence of age-dependent changes in epigenetic modifications (64, 65).

Some of our results suggest that the JAK/STAT signaling of infant CD4^+^ T cells might contribute to the Th2-biased response of infants. We observed relatively strong JAK/STAT signaling responses to the Th2 cytokine IL-4 and poorer responses to the Th1-driving cytokines IFN-γ and IL-12 in CB, and there was a statistically significant increase in the pSTAT1:pSTAT6 ratio from birth to 1 year in infant CD4^+^ T cells. However, to conclusively define the role of JAK/STAT signaling in Th cell differentiation, future studies should examine signaling events downstream of STAT activation, such as the interaction of specific STAT molecules with promoter regions of genes important in Th1 cell differentiation (e.g., tbx, il12rβ) and/or changes in epigenetic modification of these genes (Figure S4 in Supplementary Material). Furthermore, cytokine signals only represent one of the signals driving Th cell differentiation. Pathogen type, costimulatory signals by antigen-presenting cells, antigen concentration, and signals through the T cell receptor all influence CD4^+^ T cell differentiation [e.g., Ref. (19, 20, 66–78)].

The fact that the most pronounced changes for all cytokines examined occurred within the first 6 months hint at a role of common developmental factors in the regulation and maturation of the JAK/STAT signaling function in infant CD4^+^ T cells. A key factor might be the establishment of the infant’s microbiome. The importance of the maternal and infant microbiome on infant health has become the subject of intense investigation in recent years [review examples; Ref. (79–83)]. The infant requires a qualitatively distinct response from adults to allow the transition from the relatively tolerogenic milieu in the uterus to exposure to multiple environmental and pathogenic stimuli after birth. Part of this early development is the establishment of the infant’s microbiome that may come at the cost of a more suppressive or Th2-prone response. In fact, a recent study demonstrated that arginase 2-producing CD71 cells suppressed immune activation in response to host flora in neonates to allow colonization with normal flora (84). Similarly, the “layered immune system hypothesis” suggests that Tregs, induced to tolerate maternal or other antigens transmitted to the fetus *in utero*, may persist long after birth and mediate tolerogenic responses (85, 86).

The above conclusion that major changes in JAK/STAT signaling function occurred within the first 6 months does not rule out, however, that changes occurred very early, maybe even within the first month of life. Although we were able to obtain a relatively large number (*n* = 169) of healthy infant blood samples, with about equal numbers of CB samples (*n* = 70) and blood from 1-year-old infants (*n* = 64), blood samples from infants age 0.5 month to 1 year amounted to only about half this number (*n* = 35). Furthermore, <5% of all infant samples represented longitudinal samples. The sample size in our study, however, is comparable to other studies of immune ontogeny. For example, in three different studies examining the ontogeny of Toll-like receptor-mediated cytokine responses in human infants was defined using longitudinal samples from 35 (87), 30 (88), or 28 infants (89). Only two of these three studies included samples within the first year of life, with one of them sampling at 3-month intervals (88), and the other also being able to obtain samples at 2 and 6 weeks of age (89). The difficulties in obtaining samples from *healthy* infants and blood volume restrictions that limit the analysis scope of such samples constitute a main and common problem for investigators and have severely hampered the progress in our understanding of immune maturation. Despite our limitations in sample size, and by applying conservative non-parametric statistical analysis tools, we were able to, nonetheless, demonstrate broad stages of the temporal development of JAK/STAT signaling function in infant CD4^+^ T cells using. Furthermore, trends detected in the analysis of cross-sectional samples were consistent with age-dependent changes observed in longitudinal sample pairs (Figure S6 in Supplementary Material).

In summary, the current study identified age-related differences in cytokine signaling and established the time frame during which the JAK/STAT signaling function develops in infant CD4^+^ T cells in response to specific cytokines. These data provide the foundation for future studies identifying the molecular mechanisms that regulate JAK/STAT signaling in infant CD4^+^ T cells, including, but not limited to, epigenetic regulation (90), the role of miRNAs (91, 92), and the regulation of JAK kinases and STAT proteins by suppressors of cytokine signaling molecules (93, 94). These mechanistic studies were outside the scope of the current study. The data highlight how little we still understand about the multiple factors that initiate, drive, and regulate CD4^+^ T cell responses in the early postnatal period and the complex interactions between these factors. A deeper insight into postnatal development of immune cells and their functions will be instrumental for the design of novel therapeutic interventions, will inform the timely implementation of novel pediatric vaccines, and thus aid in more effectively combating childhood diseases.

## Ethics Statement

Study subjects were recruited from the University of North Carolina, Chapel Hill (UNC-CH) and the NC Children’s Hospital. Cord blood samples from full-term infants were collected immediately after birth or obtained from the Carolinas Cord Blood Bank. Direct capillary or venous blood samples (0.5–3.0 mL) from infants aged 2 weeks to 14 months were collected by nursing or phlebotomy personnel during routine physician visits. The infant participants were recruited from the North Carolina population that consists of about 46% Caucasians, 46% African-Americans, and 8% other groups, and within the ethnic categories, 10% represent Hispanics. Exclusion criteria included HIV-positive status of the mother, known in utero infections, treatment of the mother with immunosuppressive drugs, diagnosis of mother or child with immunosuppressive disorder, life-threatening malformations of the infant or life expectancy <6 months. Infant blood samples were also excluded if the infant had a bleeding disorder or had a chronic infection. The Virology, Immunology, and Microbiology Core of the UNC Center for AIDS Research provided blood samples from healthy adults. Age, sex, and race of the adult donors were unknown. The study was approved by the UNC-CH Institutional Review Board and informed parental consent was obtained. Institutional guidelines strictly adhere to the World Medical Association’s Declaration of Helsinki.

## Author Contributions

KDP, SP-W, and MP-P designed the study. JB and SP-W oversaw all clinical aspects of the study (e.g., IRB approval, consenting, sample collection). MP-P, J-R-N, RT, NC, and MM performed the experiments. MP-P, J-R-N, KM, MH, and KDP analyzed the data. MP-P, SP-W, KM, and KDP wrote the manuscript.

## Conflict of Interest Statement

The authors declare that the research was conducted in the absence of any commercial or financial relationships that could be construed as a potential conflict of interest.

## Supplementary Material

Figure S1 **Age-dependent increase in pSTAT1 activation**. Relative frequencies of naïve [CD27^+^, T(N)], central memory [CD27low, T(CM)], and effector memory/effector [CD27^−^, T(EM/Eff)] CD4^+^ T cell populations of a representative cord blood sample (top row), 1-year-old infant (middle row), and an adult (bottom row) and the corresponding histograms of pSTAT1 activation after IFN-γ stimulation (blue) are shown. pSTAT1 frequencies in unstimulated controls are shown by orange histograms. Note that *y*-axis scales differ between the age groups.

Figure S2 **Correlation between infant age and STAT activation**. From left to right, the correlations between infant age (*x*-axis) and STAT phosphorylation in response to IL-4 (pSTAT6), IFN-γ (pSTAT1), and IL-2 (pSTAT5) are graphed, with each symbol representing an individual infant and green lines representing LOESS curves with a 0.5 smooth factor. These graphs are meant to complement Table S1 in Supplementary Material and demonstrate that pSTAT6 and pSTAT1 activation plateau by about 6 months, whereas there is still a further, although slight, increase in pSTAT5 activation from 6 months to 1 year.

Figure S3 **Expression of IL-12Rb2 on CD4^+^ T cells**. **(A)** CD3^+^CD4^+^ T cells of a representative adult donor were stained for CD3^+^CD4^+^ T cells expression immediately after cell isolation (grey histogram) and after incubation for 48 h with a cytokine cocktail consisting of IL-2, IL-12, and IL-15. Note the upregulation of the IL-12Rb2 upon cytokine stimulation (blue histogram) compared to CD3^+^CD4^+^ T cells cultured in media only (orange histogram). **(B)** To validate the staining, CD3-negative cells of the same donor were analyzed for IL-12Rβ2 expression on NK cells, defined as CD3-CD14^−^CD20^−^CD56^+^. Note that IL-12Rβ2 expression, in contrast to the low expression on CD3^+^CD4^+^ T cells, was detectable on the majority of NK cells, even immediately after cell isolation. **(C)** The expression of the IL-12Rβ2 on CD3^+^CD4^+^ T cells (left histogram) and NK cells (right histogram) of a representative cord blood sample. **(D)** IL-12Rβ2 expression differs among NK cell subsets (adult donor example).

Figure S4 **Summary of age-related differences in STAT signaling and conceptual interpretation**. Median frequencies of pSTAT positive (colored circles) and negative (white) total CD4^+^ T cells expressed at fraction of median frequencies of adult CD4^+^ T cells, as measured at birth (CB, cord blood), 4–6 and 10–14 months. Each square consists of 10 × 10 circles, with each circle presenting 1%. The age is listed on the left, the cytokine and the cytokine-specific transcription factor are listed on the top of each column. IL-4-induced pSTAT6 frequencies are shown in red. The strong Th2 bias at birth is represented with the red bar/arrow on the left that is balanced with increasing age by an increasing Th1 response (green arrow on right). The most pronounced changes in STAT activation occurred in the time from birth to 6 months; in the subsequent time period from 6 months to 1 year only minor changes were noted. This is indicated by the dashed line. We propose that the establishment of the microbiome was a key factor driving these changes. In addition, other developmentally regulated factors, such as decreasing methylation levels on the *ifn*γ promoter region, likely contributed to an increase in STAT signaling. Increasing exposure to danger signals (pathogens, environmental stimuli, pediatric vaccines, etc.) drive the activation of CD4^+^ T cells and results in a changing ratio of naïve and memory CD4^+^ T cells. Activated and more differentiated CD4^+^ T cells express higher levels of certain cytokine receptors (e.g., IL-2Rα, IL-12Rβ) allowing for more effective pSTAT5 activation and signaling. We propose that at birth pSTAT5 may predominantly activate the il4 and il4ra genes, while with increasing age pSTAT5 will be able to activate to also activate *ifn*γ and *il12rβ* genes and thereby, promote Th1 responses. This figure is meant to summarize our data and present some factors that may have contributed to the observed age-related changes in JAK/STAT signaling function and present a potential role of IL-2 in driving Th1 development in infants. Other factors are likely important in postnatal development of JAK/STAT signaling; this figure is by no means exhaustive. The complex interplay between these factors and other immune cells was outside the scope of the current study.

Figure S5 **Changes in STAT activation in infant CD4^+^ T cell subpopulation from birth to 1 year**. Analogous to Figure S4 in Supplementary Material, median frequencies of pSTAT positive (colored circles) and negative (white) naïve [T(N)], central memory [T(CM)], and effector memory/effector [T(EM/Eff)] CD4^+^ T cells are expressed at fraction of median frequencies of adult CD4^+^ T cell subpopulations at birth (CB, cord blood) and 1 year of age (10–14 months). Each square consists of 10 × 10 circles, with each circle presenting 1%. The CD4^+^ T cell subpopulations are listed on the column top and the cytokine with its relevant transcription factor are listed on the left. The color coding is as described in Figure S4 in Supplementary Material.

Figure S6 **Age-dependent changes in STAT activation in longitudinal infant blood samples**. **(A)** The frequencies of pSTAT6, pSTAT1, and pSTAT5^+^ CD4^+^ T cells after stimulation of longitudinal samples from the same infant with IL-4, IFN-γ, or IL-2, respectively. Samples from the same infant are represented by the same symbol and longitudinal data points are connected by a black line. **(B)** Representative histograms of samples shown in panel **(A)** are depicted.
