# Supplementary material for: Increasing JAK/STAT Signaling Function of Infant CD4+ T Cells during the First Year of Life
Source: Front Pediatr. 2017 Feb 21;5:15. doi: 10.3389/fped.2017.00015 (PMC5318443; doi:10.3389/fped.2017.00015)
Supplement: Supplementary file 1 [file Table_1.pdf]

Table S1: Age-dependent changes in STAT activation

Cytokine/STAT	Cell Population	Time Period (Months)	Spearman Correlation rho	p-value
IL-4/ pSTAT6	CD4$^+$T	0-14	0.6075	<0.0001
	CD4$^+$T (N)		0.6415	<0.0001
	CD4$^+$T (CM)		0.4465	0.0012
	CD4$^+$T(EM/Eff)		0.7297	<0.0001
IFN-γ/ pSTAT1	CD4$^+$T	0-14	0.7919	<0.0001
	CD4$^+$T (N)		0.7869	<0.0001
	CD4$^+$T (CM)		0.7247	<0.0001
	CD4$^+$T(EM/Eff)		0.6682	<0.0001
IL-2/ pSTAT5	CD4$^+$T	0-14	0.8197	<0.0001
	CD4$^+$T (N)		0.8040	<0.0001
	CD4$^+$T (CM)		0.8246	<0.0001
	CD4$^+$T(EM/Eff)		0.8273	<0.0001
IL-4/ pSTAT6	CD4$^+$T	0-6	0.5240	0.0006
	CD4$^+$T (N)		0.5385	<0.0003
	CD4$^+$T (CM)		0.3387	0.0349
	CD4$^+$T(EM/Eff)		0.5608	0.0003
IFN-γ/ pSTAT1	CD4$^+$T	0-6	0.7193	<0.0001
	CD4$^+$T (N)		0.7625	<0.0001
	CD4$^+$T (CM)		0.7427	<0.0001
	CD4$^+$T(EM/Eff)		0.5793	<0.0001
IL-2/ pSTAT5	CD4$^+$T	0-6	0.6421	<0.0001
	CD4$^+$T (N)		0.6683	<0.0001
	CD4$^+$T (CM)		0.6654	<0.0001
	CD4$^+$T(EM/Eff)		0.6957	<0.0001
IL-4/ pSTAT6	CD4$^+$T	7-14	-0.0766	0.8643
IFN-γ/ pSTAT1	CD4$^+$T	7-14	-0.4206	0.0457
IL-2/ pSTAT5	CD4$^+$T	7-14	0.0000	>0.9999
