# Supplementary material for: Increasing JAK/STAT Signaling Function of Infant CD4+ T Cells during the First Year of Life
Source: Front Pediatr. 2017 Feb 21;5:15. doi: 10.3389/fped.2017.00015 (PMC5318443; doi:10.3389/fped.2017.00015)
Supplement: Figure S1 — Age-dependent increase in pSTAT1 activation. Relative frequencies of naïve [CD27+, T(N)], central memory [CD27low, T(CM)], and effector memory/effector [CD27−, T(EM/Eff)] CD4+ T cell populations of a representative cord blood sample (top row), 1-year-old infant (middle row), and an adult (bottom row) and the corresponding histograms of pSTAT1 activation after IFN-γ stimulation (blue) are shown. pSTAT1 frequencies in unstimulated controls are shown by orange histograms. Note that y-axis scales differ between the age groups. [file Image_1.pdf]

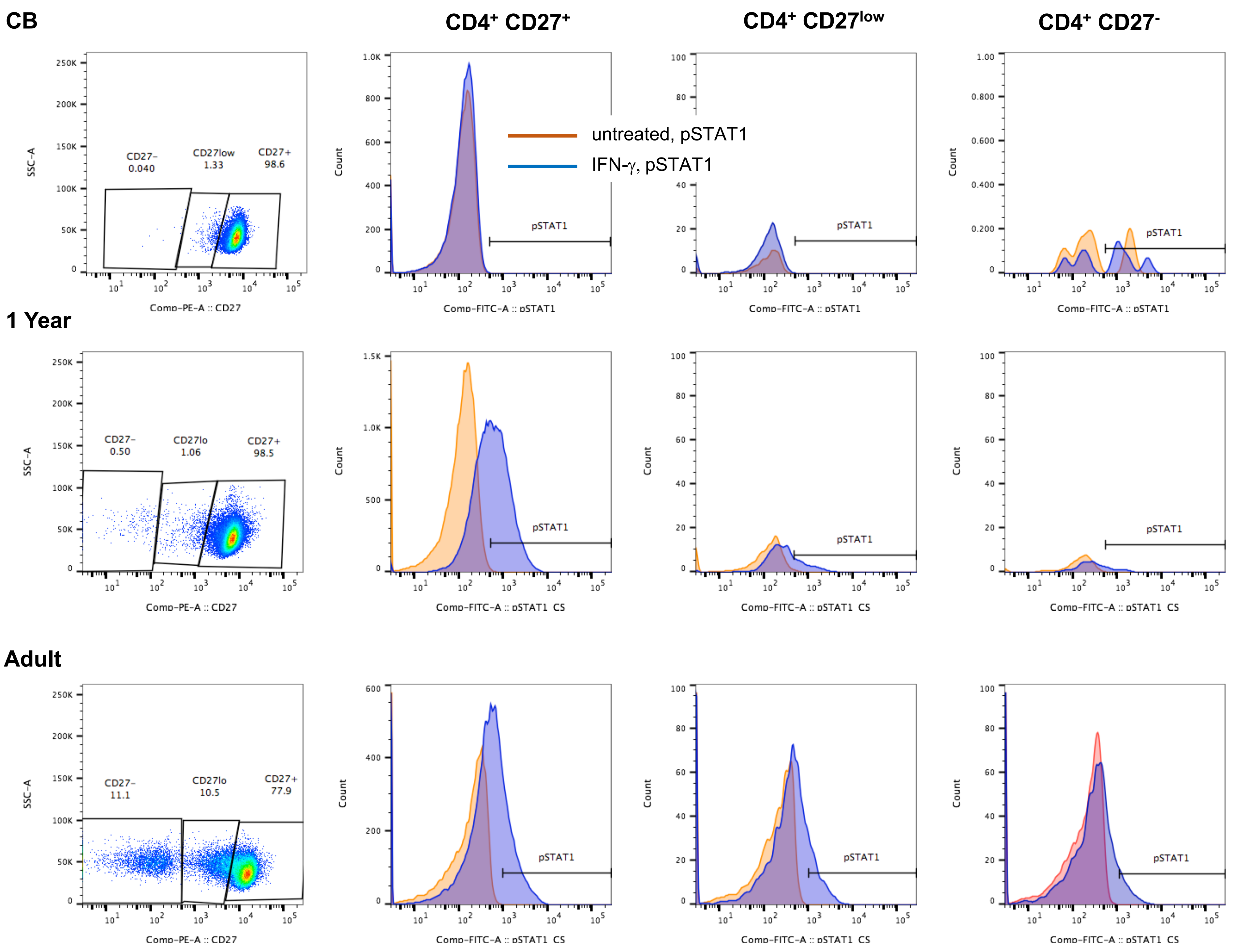


Figure S1, dela Pena-Ponce et al.
