# Supplementary figures and images for: Increasing JAK/STAT Signaling Function of Infant CD4+ T Cells during the First Year of Life
Source: Front Pediatr. 2017 Feb 21;5:15. doi: 10.3389/fped.2017.00015 (PMC5318443; doi:10.3389/fped.2017.00015)

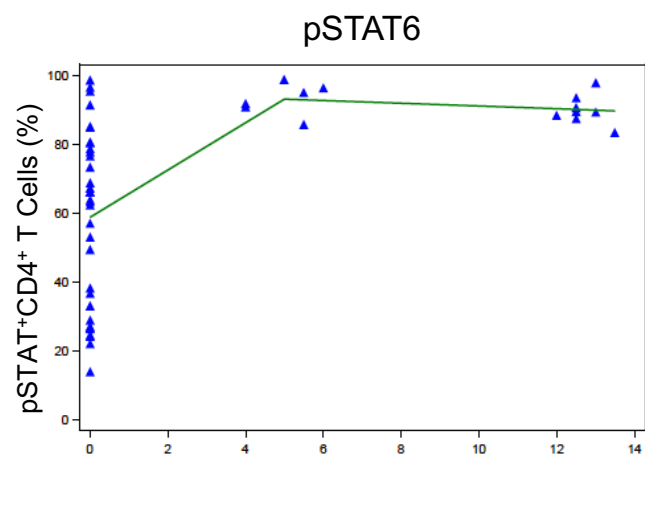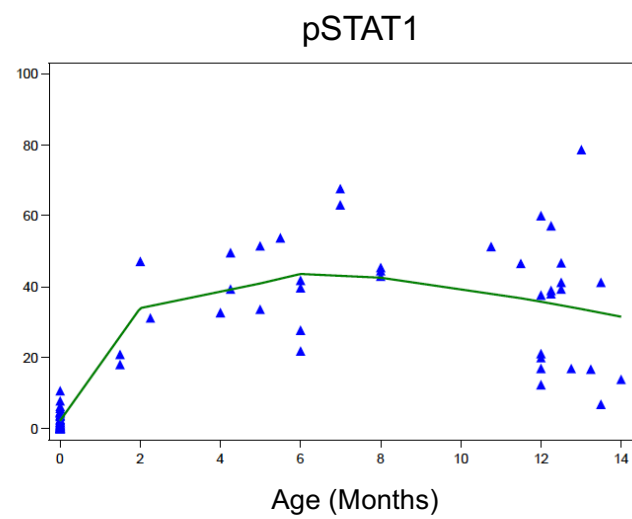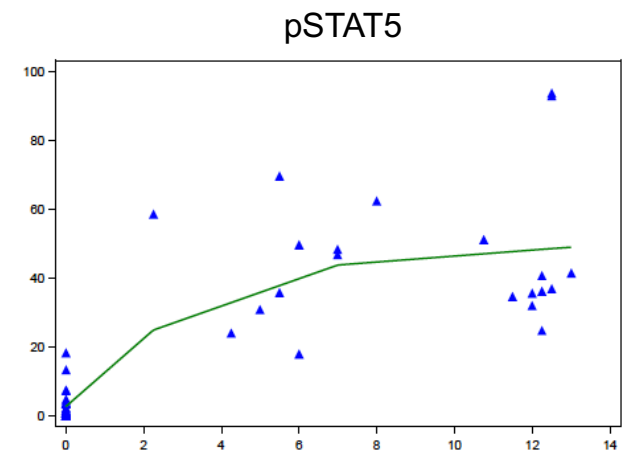

Figure S2, dela Pena-Ponce et al.

Supplement: Figure S2 — Correlation between infant age and STAT activation. From left to right, the correlations between infant age (x-axis) and STAT phosphorylation in response to IL-4 (pSTAT6), IFN-γ (pSTAT1), and IL-2 (pSTAT5) are graphed, with each symbol representing an individual infant and green lines representing LOESS curves with a 0.5 smooth factor. These graphs are meant to complement Table S1 in Supplementary Material and demonstrate that pSTAT6 and pSTAT1 activation plateau by about 6 months, whereas there is still a further, although slight, increase in pSTAT5 activation from 6 months to 1 year. [file Image_2.pdf]

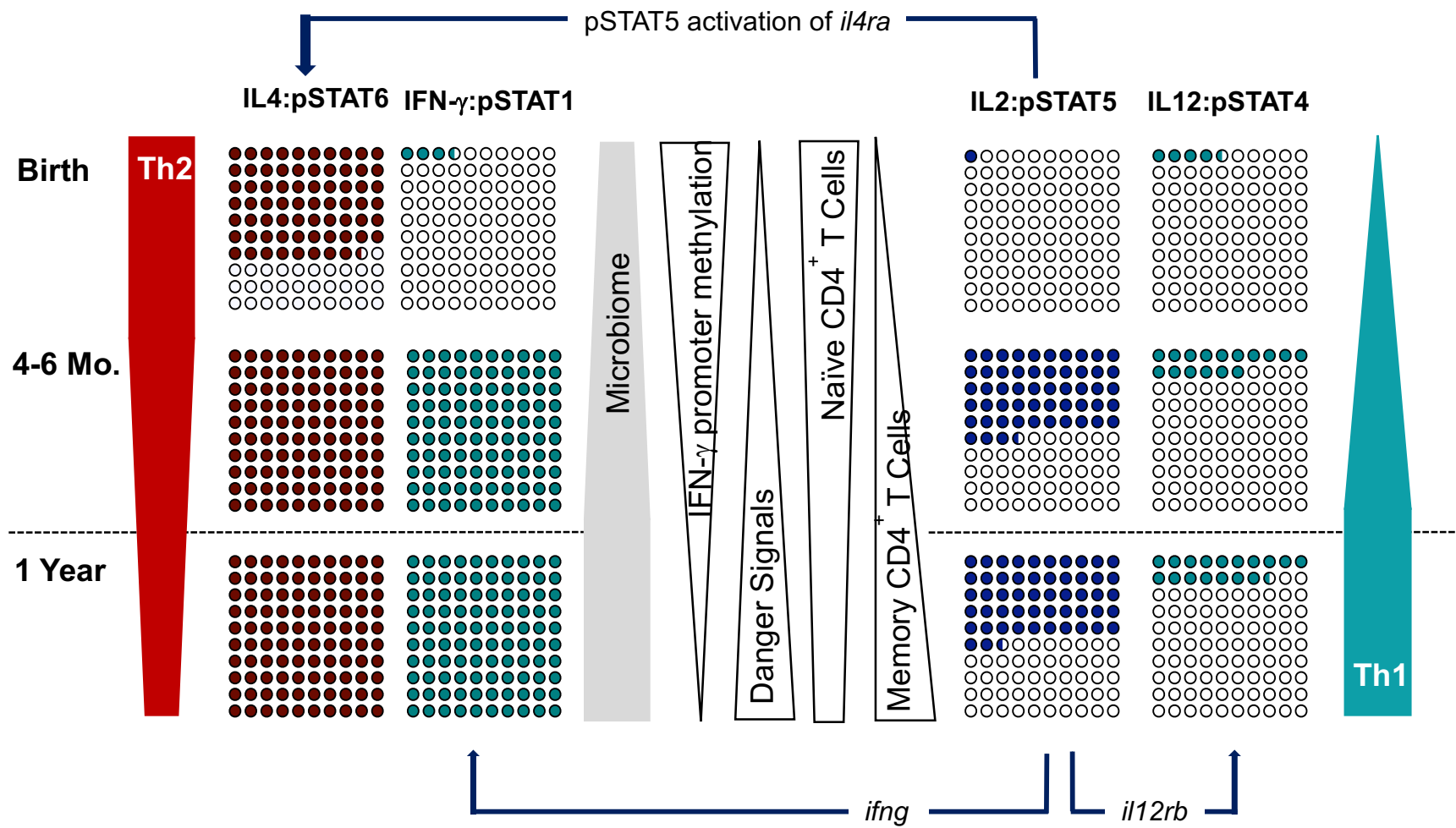

Figure S4  
 dela Pena-Ponce et al.

Supplement: Figure S4 — Summary of age-related differences in STAT signaling and conceptual interpretation. Median frequencies of pSTAT positive (colored circles) and negative (white) total CD4+ T cells expressed at fraction of median frequencies of adult CD4+ T cells, as measured at birth (CB, cord blood), 4–6 and 10–14 months. Each square consists of 10 × 10 circles, with each circle presenting 1%. The age is listed on the left, the cytokine and the cytokine-specific transcription factor are listed on the top of each column. IL-4-induced pSTAT6 frequencies are shown in red. The strong Th2 bias at birth is represented with the red bar/arrow on the left that is balanced with increasing age by an increasing Th1 response (green arrow on right). The most pronounced changes in STAT activation occurred in the time from birth to 6 months; in the subsequent time period from 6 months to 1 year only minor changes were noted. This is indicated by the dashed line. We propose that the establishment of the microbiome was a key factor driving these changes. In addition, other developmentally regulated factors, such as decreasing methylation levels on the ifnγ promoter region, likely contributed to an increase in STAT signaling. Increasing exposure to danger signals (pathogens, environmental stimuli, pediatric vaccines, etc.) drive the activation of CD4+ T cells and results in a changing ratio of naïve and memory CD4+ T cells. Activated and more differentiated CD4+ T cells express higher levels of certain cytokine receptors (e.g., IL-2Rα, IL-12Rβ) allowing for more effective pSTAT5 activation and signaling. We propose that at birth pSTAT5 may predominantly activate the il4 and il4ra genes, while with increasing age pSTAT5 will be able to activate to also activate ifnγ and il12rβ genes and thereby, promote Th1 responses. This figure is meant to summarize our data and present some factors that may have contributed to the observed age-related changes in JAK/STAT signaling function and present a potential ro [file Image_4.pdf]

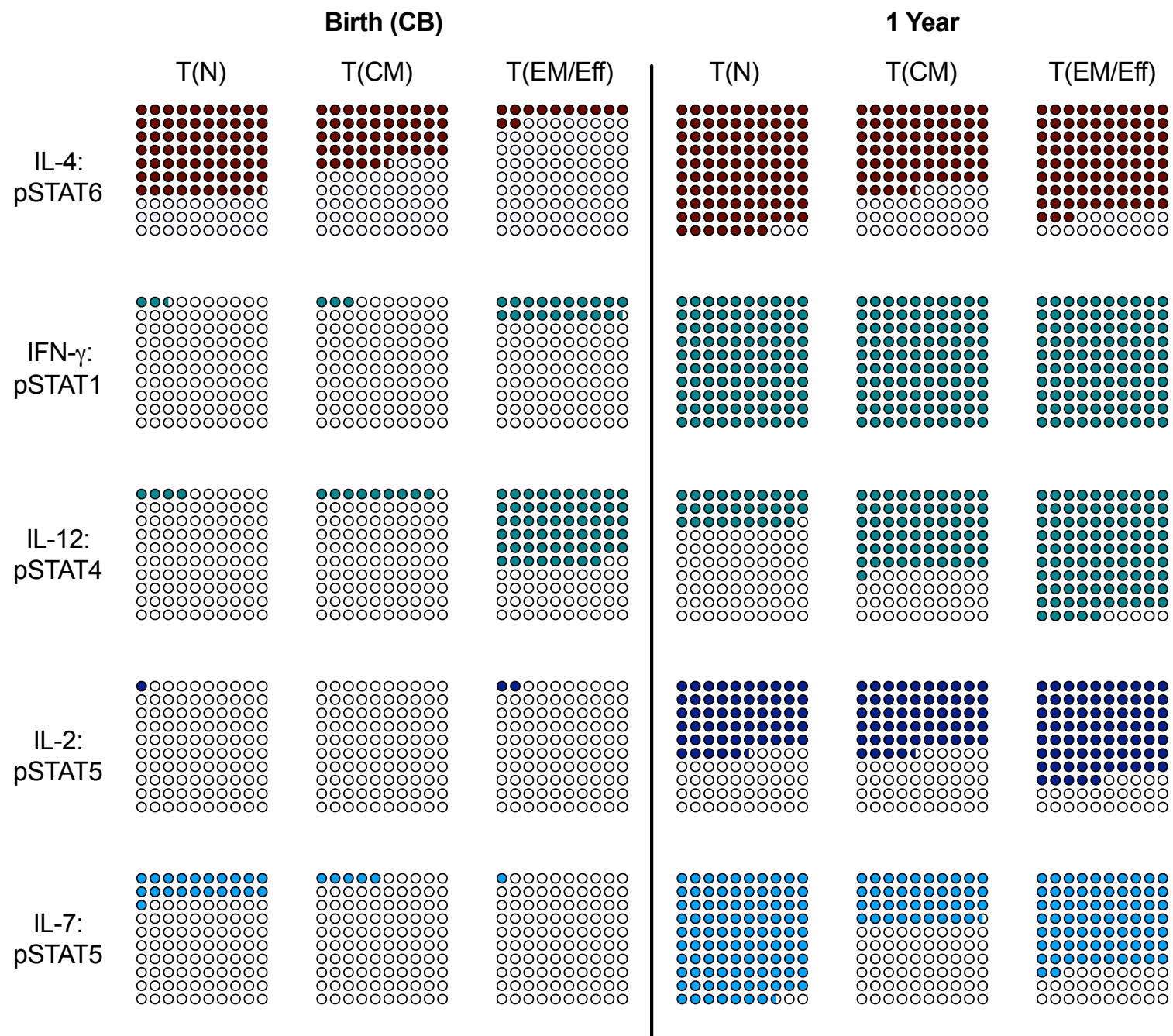

Figure S5, dela Pena-Ponce et al.

Supplement: Figure S5 — Changes in STAT activation in infant CD4+ T cell subpopulation from birth to 1 year. Analogous to Figure S4 in Supplementary Material, median frequencies of pSTAT positive (colored circles) and negative (white) naïve [T(N)], central memory [T(CM)], and effector memory/effector [T(EM/Eff)] CD4+ T cells are expressed at fraction of median frequencies of adult CD4+ T cell subpopulations at birth (CB, cord blood) and 1 year of age (10–14 months). Each square consists of 10 × 10 circles, with each circle presenting 1%. The CD4+ T cell subpopulations are listed on the column top and the cytokine with its relevant transcription factor are listed on the left. The color coding is as described in Figure S4 in Supplementary Material. [file Image_5.pdf]

A

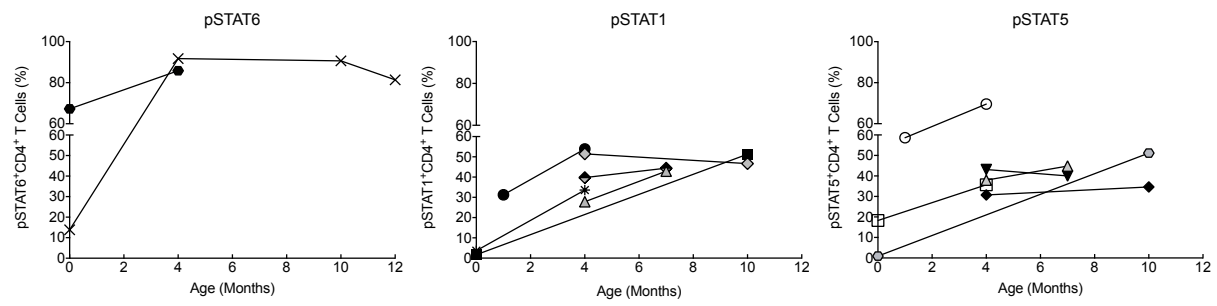

B

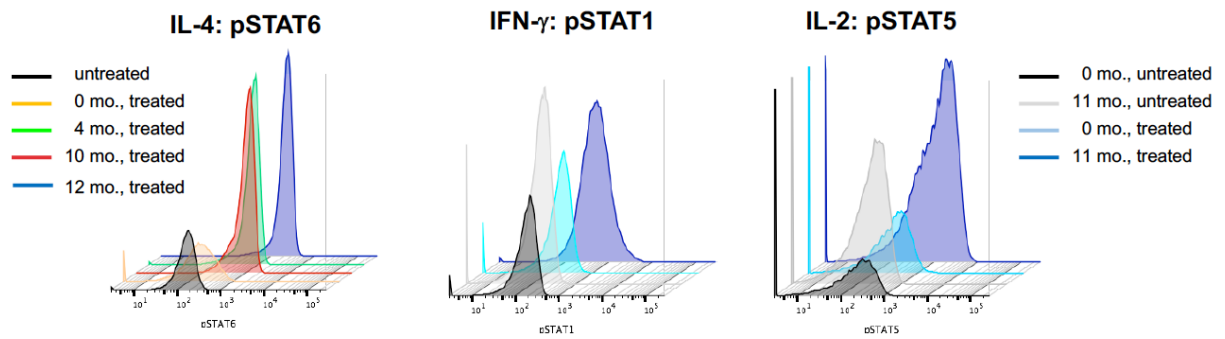

Figure S6  
dela Peña-Ponce et al.

Supplement: Figure S6 — Age-dependent changes in STAT activation in longitudinal infant blood samples. (A) The frequencies of pSTAT6, pSTAT1, and pSTAT5+ CD4+ T cells after stimulation of longitudinal samples from the same infant with IL-4, IFN-γ, or IL-2, respectively. Samples from the same infant are represented by the same symbol and longitudinal data points are connected by a black line. (B) Representative histograms of samples shown in panel (A) are depicted. [file Image_6.pdf]
