# Supplementary material for: Increasing JAK/STAT Signaling Function of Infant CD4+ T Cells during the First Year of Life
Source: Front Pediatr. 2017 Feb 21;5:15. doi: 10.3389/fped.2017.00015 (PMC5318443; doi:10.3389/fped.2017.00015)
Supplement: Figure S3 — Expression of IL-12Rb2 on CD4+ T cells. (A) CD3+CD4+ T cells of a representative adult donor were stained for CD3+CD4+ T cells expression immediately after cell isolation (grey histogram) and after incubation for 48 h with a cytokine cocktail consisting of IL-2, IL-12, and IL-15. Note the upregulation of the IL-12Rb2 upon cytokine stimulation (blue histogram) compared to CD3+CD4+ T cells cultured in media only (orange histogram). (B) To validate the staining, CD3-negative cells of the same donor were analyzed for IL-12Rβ2 expression on NK cells, defined as CD3-CD14−CD20−CD56+. Note that IL-12Rβ2 expression, in contrast to the low expression on CD3+CD4+ T cells, was detectable on the majority of NK cells, even immediately after cell isolation. (C) The expression of the IL-12Rβ2 on CD3+CD4+ T cells (left histogram) and NK cells (right histogram) of a representative cord blood sample. (D) IL-12Rβ2 expression differs among NK cell subsets (adult donor example). [file Image_3.pdf]

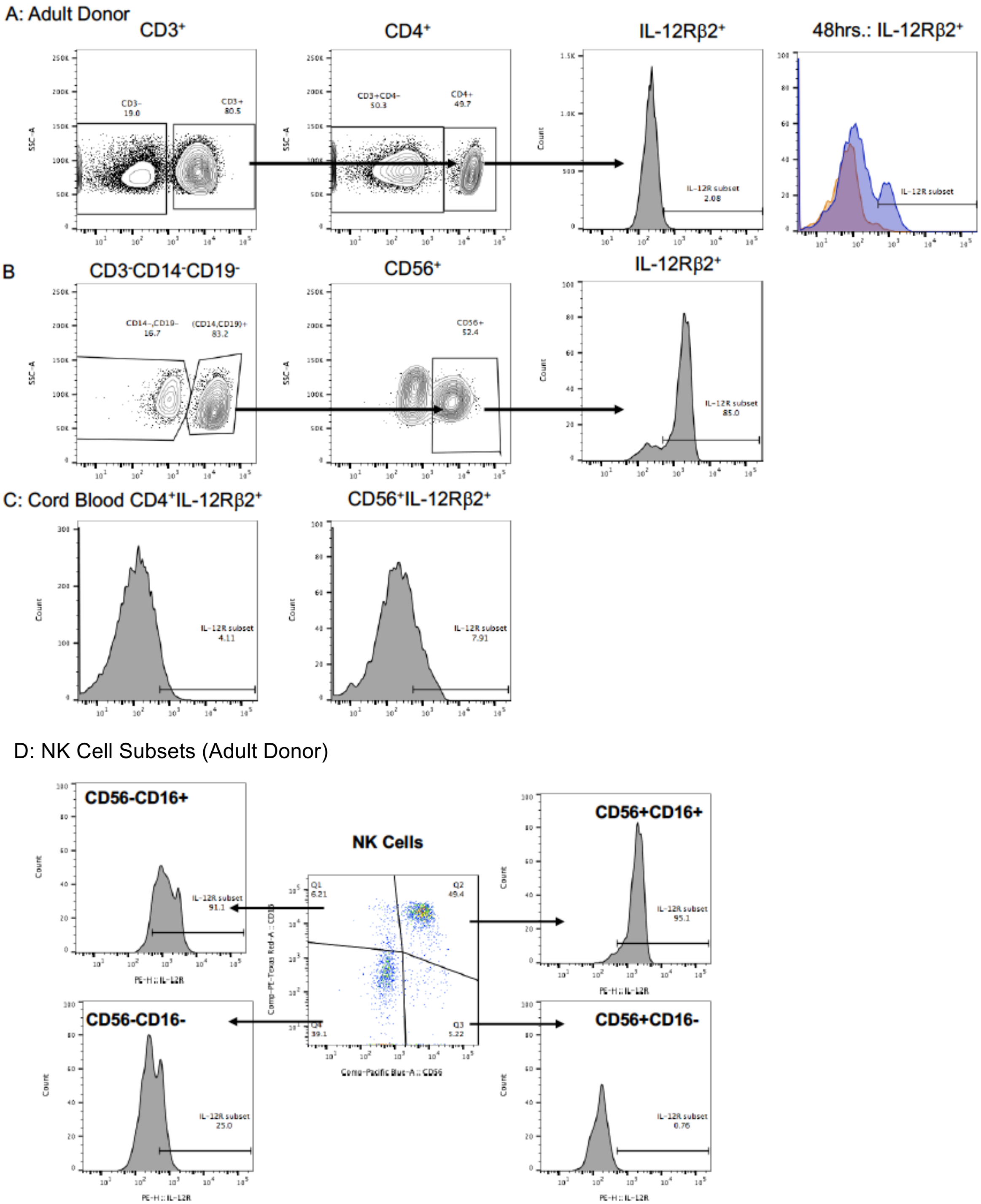


Figure S3, dela Pena-Ponce et al.
